# Graph neural networks in Alzheimer's disease diagnosis: a review of unimodal and multimodal advances

**DOI:** 10.3389/fnins.2025.1623141

**Published:** 2025-09-26

**Authors:** Shahzad Ali, Michele Piana, Matteo Pardini, Sara Garbarino

**Affiliations:** ^1^Department of Pharmacy and Biotechnology, Alma Mater Studiorum - Universitá di Bologna, Bologna, Italy; ^2^Life Science Computational Laboratory (LISCOMP), Istituto di Ricovero e Cura a Carattere Scientifico (IRCCS) Ospedale Policlinico San Martino, Genova, Italy; ^3^Department of Information Sciences, University of Education, Lahore, Pakistan; ^4^Dipartimento di Matematica, Universitá degli Studi di Genova, Genova, Italy; ^5^Dipartimento di Neuroscienze, Riabilitazione, Oftalmologia, Genetica e Scienze Materno-Infantili, Università degli Studi di Genova, Genova, Italy; ^6^Istituto di Ricovero e Cura a Carattere Scientifico (IRCCS) Ospedale Policlinico San Martino, Genova, Italy

**Keywords:** Alzheimer's disease, deep learning, diagnosis, graph neural network, multimodal, neuroimaging, neurological disorders, review

## Abstract

Alzheimer's Disease (AD), a leading neurodegenerative disorder, presents significant global health challenges. Advances in graph neural networks (GNNs) offer promising tools for analyzing multimodal neuroimaging data to improve AD diagnosis. This review provides a comprehensive overview of GNN applications in AD diagnosis, focusing on data sources, modalities, sample sizes, classification tasks, and diagnostic performance. Drawing on extensive literature searches across PubMed, IEEE Xplorer, Scopus, and Springer, we analyze key GNN frameworks and critically evaluate their limitations, challenges, and opportunities for improvement. In addition, we present a comparative analysis to evaluate the generalizability and robustness of GNN methods across different datasets, such as ADNI, OASIS, TADPOLE, UK Biobank, in-house, etc. Furthermore, we provide a critical methodological comparison across families of GNN architectures (i.e., GCN, ChebNet, GraphSAGE, GAT, GIN, etc.) in the context of AD. Finally, we outline future research directions to refine GNN-based diagnostic methods and highlight their potential role in advancing AI-driven neuroimaging solutions. Our findings aim to foster the integration of AI technologies in neurodegenerative disease research and clinical practice.

## 1 Introduction

Neurodegenerative disorders (NDDs), such as Alzheimer's disease (AD), represent a significant global public health challenge. AD is a progressive condition, due to the presence of a progressive accumulation of misfolded proteins, that disrupts individuals' daily functioning, with hallmark symptoms including cognitive decline, memory impairment, and emotional instability (Zhang et al., 2023e; Liu et al., 2023a; Association et al., 2020; Zhang et al., 2023d). As the most common form of irreversible dementia, AD predominantly affects individuals over the age of 65. Its diagnosis remains challenging due to the multiple causes of dementia beyond AD (Tăuţan et al., 2021).

From a clinical point of view, AD subjects can present in the dementia phase (i.e., with significant cognitive deficits that impact daily activities) or in the mild cognitive impairment (MCI) phase (i.e., with subtle cognitive deficits that do not impact daily activities). Subjects with MCI often progress to over dementia, with studies of subjects in the AD continuum estimating that over half of MCI cases advance to dementia during clinical observation (Zhang et al., 2023e; Liu et al., 2023a; Association et al., 2020; Zhang et al., 2023d). Early and accurate diagnosis of AD, especially at the MCI stage, is crucial for timely interventions and improved patient outcomes.

AD diagnosis traditionally involves clinical examinations, behavioral assessments, fluid biomarkers, and neuroimaging techniques. Advances in neuroimaging modalities such as magnetic resonance imaging (MRI), diffusion tensor imaging (DTI), functional MRI (fMRI), and positron emission tomography (PET) have significantly enhanced the potential for accurate screening, diagnosis, and prognosis (Kipf and Welling, 2016; Zhou et al., 2022a). More recently, the integration of deep learning (DL) techniques with neuroimaging data has demonstrated considerable promise in improving diagnostic precision (Meng and Zhang, 2023). Computer-aided diagnostic (CAD) systems leveraging neuroimaging and DL methods have gained attention for their ability to assist in AD detection (Zhou et al., 2022a). Traditional DL architectures, such as multilayer perceptron (MLP), convolutional neural networks (CNNs), and recurrent neural networks (RNNs), have been applied to analyze disorders linked to cognitive decline (Zong and Wang, 2023). While CNNs are frequently employed in CAD systems, they encounter significant challenges in handling multimodal neuroimaging data. These limitations include their inability to account for inter-subject correlations, limited interpretability when integrating multimodal data, and requirements for uniform input dimensions across channels (Zhang et al., 2023e). Furthermore, since AD leads to structural and functional changes in brain connectivity, conventional DL approaches like CNNs struggle to effectively capture the network-like properties of brain data. Graph-based methods have been introduced to address these challenges by modeling brain connectivity, subnetworks, and local interactions in a more biologically relevant manner (Pasquini et al., 2015). These approaches provide a pathway to better understand the intricate dynamics underlying AD progression and may overcome some limitations of traditional DL models.

Graph neural networks (GNNs) extend convolutional neural networks (CNNs) to non-Euclidean domains by incorporating graph structures and propagating information between connected nodes (Zhang et al., 2023e; Kipf and Welling, 2016). This capability makes GNNs particularly suitable for applications such as AD diagnosis, where data often include complex multimodal relationships. GNNs support two primary approaches for graph-based representation in neuroimaging studies: (1) subject-level graphs, where brain regions of interest (ROIs) are treated as nodes, and the structural or functional connectivity between brain ROIs are represented as edges; and (2) population graphs, where individual subjects serve as nodes, and edges encode relationships based on demographic data, imaging modalities or other features such as genetic or behavioral similarities (Tekkesinoglu and Pudas, 2024; Zhou et al., 2020). Among GNN variants, graph convolutional networks (GCNs) have been widely employed for medical applications (Zhang et al., 2023b). GCNs operate directly on graph data, leveraging the topological and relational information within the graph structure (Zhang et al., 2023e; Kipf and Welling, 2016). For instance, in subject-level graphs, nodes represent brain ROIs, while edges capture anatomical or functional connectivity. GCNs aggregate and filter features from neighboring nodes, thereby generating enriched node-level feature representations that enhance disease prediction and support graph-level analyses (Parisot et al., 2018). This ability to integrate and process multimodal information within a flexible graph framework positions GNNs as a powerful tool for advancing our understanding of complex conditions like AD.

While several reviews have discussed the role of GNNs in disease diagnosis, their specific applications in AD remain underexplored. For instance, Ahmedt-Aristizabal et al. (2021) reviewed GNNs for general disease diagnosis, while Zhang et al. (2023c) examined their use in brain imaging for neurodegenerative disorders. Similarly, Zhang et al. (2023b) provided a systematic review of GNN-based approaches for image-guided diagnosis. Despite these efforts, existing reviews often lack in-depth analysis of GNN applications that integrate multimodal neuroimaging data, an essential aspect for accurate early diagnosis of AD. In addition, recent advances in GNN methods and their advantages over traditional unimodal approaches are underrepresented in these discussions. This review aims to address these gaps by offering a comprehensive overview of GNN applications for the diagnosis of AD, with a particular emphasis on unimodal and multimodal neuroimaging advances in the domain of AD. The analysis highlights the potential benefits of multimodal approaches compared to unimodal techniques, with the aim of demonstrating how GNN can provide novel insights and improve diagnostic outcomes in AD research.

The rest of this review article is organized as follows: Section 2 outlines the methodology, detailing the literature search strategy and publication selection process. Section 3 provides an overview of GNNs and their framework in the context of AD diagnosis. Section 4 presents a detailed review of GNN applications for AD diagnosis, with comparisons between unimodal and multimodal data approaches. Section 5 discusses the key challenges, limitations, and potential future directions in GNN research for AD. Finally, our conclusions are offered in Section 6.

## 2 Methodology

### 2.1 Data sources and literature search queries

We conducted an extensive search across databases, such as PubMed, Science Direct, Scopus, and IEEE Xplore in August 2024 to identify high-quality, highly cited studies using GNNs and neuroimaging for AD diagnosis and prediction. Our search focused on original research publications that employed graph neural network methodologies for neuroimaging-based AD diagnosis. To ensure relevant results, we used carefully selected keywords and search queries (SQs), including:

- SQ-1: (Alzheimer's disease) AND (Multimodal data) AND (Graph Neural Network)).
- SQ-2: (Alzheimer's disease) AND (Neuroimaging) AND (Graph Neural Network)).
- SQ-3: (Alzheimer's disease) AND (Multimodal data) AND (Brain connectivity) AND (Deep Learning).
- SQ-4: (Alzheimer's disease) AND (Graph Neural Network) AND ((MRI) OR (fMRI) OR (DTI) OR (PET)).

We also used “graph convolutional network” as an alternative to “graph neural network,” and considered common abbreviations such as “DL” for “deep learning” and the full names for imaging modalities such as MRI, DTI, fMRI, and PET.

**Figure 1:**
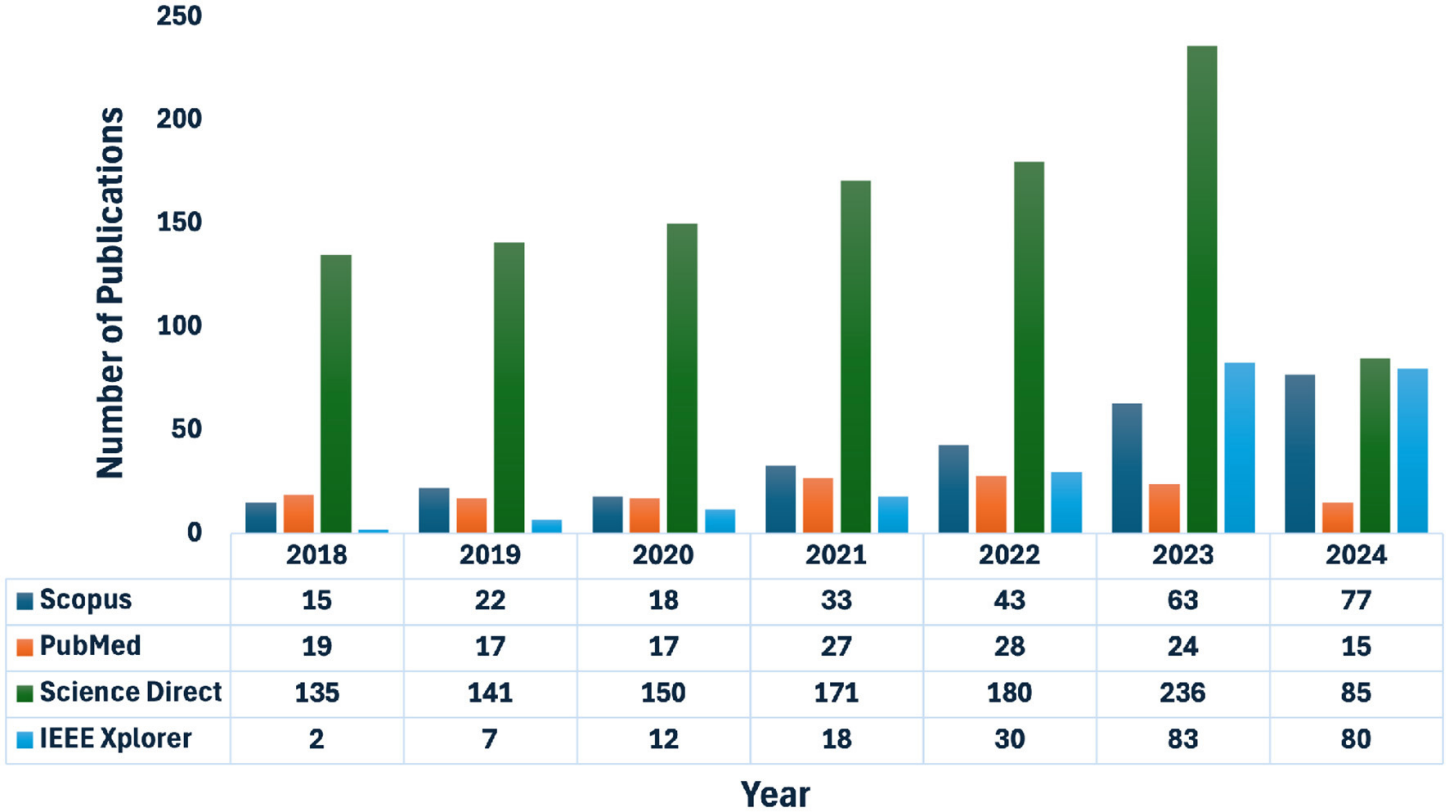
Article retrieved from different databases and sources.

As a result, a total of 1,748 publications have been retrieved from the aforementioned databases using these SQs. Figure 1 illustrates the year-wise distribution of publications obtained from these databases by applying SQ-1 to SQ-4.

### 2.2 Publication selection process

**Figure 2:**
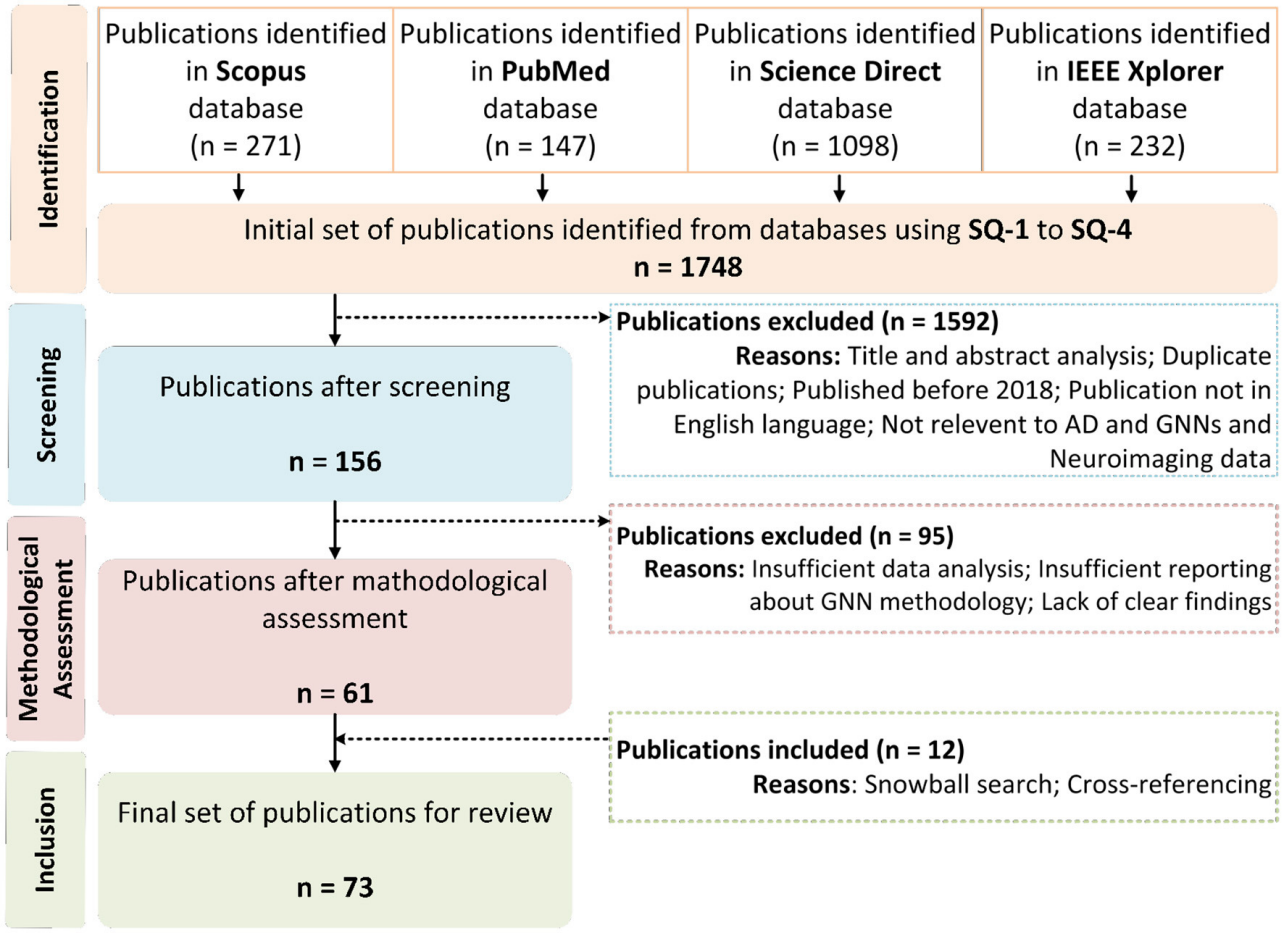
The flowchart of the publication selection process.

The initial database search yielded numerous duplicate and irrelevant publications. To refine the selection process for this study, we established specific inclusion and exclusion criteria aimed at retaining only relevant studies. Publications that did not meet these criteria were excluded, leading to the identification of the most pertinent studies, as outlined in the flowchart in Figure 2.

**Exclusion criteria (screening):**

i. Duplicate publications retrieved from different databases.
ii. Publications in languages other than English.
iii. Publications published before 2018.
iv. Conference abstracts, surveys, reviews, and theses.
v. Publications unrelated to the application of GNN methods in AD diagnosis.
vi. Publications lacking sufficient detail on GNN models, such as training procedures, architecture, or evaluation criteria applied to AD diagnosis.

**Inclusion criteria (methodological assessment):**

i. Full-text publications only.
ii. Publications focusing on AD diagnosis using GNNs.
iii. Publications utilizing neuroimaging data.
iv. Publications addressing the interpretability and explainability of GNNs.
v. Publications identified through snowball searches and cross-referencing.

Figure 1 depicts the comprehensive search process across databases and search engines, which identified a total of 1,748 publications. Figure 2 outlines the subsequent steps of screening, methodological assessments, cross-referencing, and snowball searches, which resulted in the inclusion of 73 publications for in-depth review, each reporting on the use of GNN methods combined with neuroimaging data to diagnose AD and monitor its progression. All selected publications were thoroughly examined, with data extracted and analyzed to provide an overall picture of the reviewed studies (Tables 1, 2). The extracted information included references, publication years, data sources (i.e., databases), number of subjects, subject distribution, specific modalities, and AD classification (binary or multilevel), providing a structured overview of the collected data.

**Table 1:** Summary of key findings of the papers using GNNs and Unimodal biomarkers for AD diagnosis.

**Sr**.	**Article**	**Year**	**Database (size)**	**Modalities**	**Subjects**			**GNN Architecture**	**Classification Accuracy (%)**				
					***NC***	***MCI***	***AD***	***FE-Graph Convolution (Pooling)***	***NC/AD***	***sMCI/pMCI***	***NC/MCI***	***MCI/AD***	***Multilevel***
1	(Wee et al., 2019)	2019	ADNI-1 (1012)	MRI	242	415	355	Spectral-ChebNet (Global Average Pooling)	85.8	60.9^*cc*^	69.3^*ca*^, 51.8^*cb*^	79.2^*cd*^, 65.2^*ce*^	–
			ADNI-2 (1083)		300	314/218^*db*^	261						
			in-house (347)		176	128	43						
2	(Kim et al., 2021)	2021	ADNI (806)	MRI	266	312	228	Spectral-GCN + Temporal LSTM (Global Average Pooling)	–	–	–	53.5	–
3	(Zhu et al., 2022b)	2022	ADNI (202)	MRI	52	56/43^*da*^	51	Spectral-GCN (–)	–	–	–	–	96
4	(Fan et al., 2022)	2022	ADNI (1,253)	MRI	330	296/248^*da*^	336	Spatial-Other (Global Summation Pooling)	89.0^*ba*^	71.90^*ba*^	–	–	53.74
			OASIS (193)		93	–	100		67.22^*ba*^	–	–	–	–
			NIFD (327)		114	–	213		83.27^*ba*^	–	–	–	–
5	(Peng et al., 2022)	2022	ADNI (911)	MRI	–	536	375	Spectral-GCN (–)	–	–	–	75.8	–
6	(Zhang et al., 2023a)	2023	ADNI (1,644)	MRI	459	768	417	Spectral-GCN (–)	92.57	–	73.77	–	63.23
7	(Fan et al., 2023)	2023	ADNI (666)	MRI	330	–	336	Spectral-Other (–)	86.20^*^	–	–	–	–
8	(Aafiya and Jeyachidra, 2024)	2024	Kaggle	MRI	–	–	–	Spectral-GCN (–)	96.18	–	–	–	–
9	(Liu et al., 2024)	2024	ADNI (107)	MRI	48	–	59	Spectral GCN (Global Average Pooling)	89.76	–	–	–	–
10	(Hao et al., 2024)	2024	ADNI (518)	MRI	170	125	223	Spectral GCN (–)	90.18	–	76.1	84.07	–
11	(Ktena et al., 2018)	2018	UK Biobank (500)	fMRI	–	–	–	Spectral-ChebNet (global summation pooling)	–	–	70.4	–	–
12	(Zhao et al., 2019)	2019	ADNI (184)	rs-fMRI	67	77/40^*db*^	–	Spectral-Other (–)	–	85.6^*cc*^	78.4^*ca*^, 84.3^*cb*^	–	–
13	(Liu X. et al., 2020)	2020	ADNI (133)	rs-fMRI	–	99	34	Spectral-GCN (–)	–	–	–	77.2	–
14	(Yao et al., 2021)	2021	ADNI (367)	fMRI	179	191	–	MultiGraph-GCN (–)	–	–	83.4	–	–
15	(Gu et al., 2021)	2021	ADNI (311)	fMRI	106	95	110	Spectral-GCN (–)	89.9	–	88.9	94.7	–
16	(Lee et al., 2021)	2021	ADNI (101)	fMRI	48	53^*dc*^	–	Spectral-GCN (Global Maximum Pooling)	–	–	74.42^*ca*^	–	–
17	(Kumar et al., 2022)	2022	ADNI (189)	fMRI	52	59/45^*db*^	33	Spectral-ChebNet (–)	–	–	–	81.8	–
18	(Wang X. et al., 2023)	2023	OASIS (1,000)	fMRI	484	–	516	ST-Other (Global Maximum Pooling)	99.16	–	–	–	–
19	(Tang et al., 2022)	2022	OASIS (1,326)	fMRI	–	–	–	Spectral-GCN (Hierarchical TopK Pooling)	77.51	–	–	–	–
20	(Mei et al., 2022)	2022	ADNI (483)	fMRI	565	345^*dc*^	–	Spectral-GCN (Hierarchical Diff Pooling)	–	–	73.37^*ca*^	–	–
21	(Wen et al., 2022)	2022	ADNI (133)	rs-fMRI	–	99	34	Spectral-GCN (Hierarchical Eigen Pooling)	–	–	–	69.38	–
22	(Qin et al., 2022)	2022	ADNI (91)	rs-fMRI	47	–	44	Spectral-GCN (Global Average Pooling)	83.3	–	–	–	–
23	(Liu et al., 2023b)	2023	ADNI, OASIS, ABIDE, in-house (4410)	fMRI	2512	–	1,898^*dm*^	Spectral-GCN (–)	71.3	–	–	–	–
24	(Zuo and Kamata, 2023)	2023	ADNI (72)	fMRI	30	26	16	Spectral-GCN (–)	80	–	–	–	71.43
25	(Liu et al., 2023c)	2023	ADNI (635)	fMRI	366	269	–	Spectral-GCN (–)	–	–	73.54	–	–
26	(Wang Z. et al., 2023)	2023	ADNI (330)	fMRI	185	–	118	Spatial-GIN (Global Pooling)	90.44	–	–	–	–
27	(Liu et al., 2023a)	2023	ADNI (531)	rs-fMRI	364	117	50	ST-RNN (Hierarchical Pooling)	79.1	–	–	–	–
			OASIS (250)		207	–	43		88.9	–	78.6	–	–
			ABIDE (1011)		512	–	499		72.7	–	–	–	–
28	(Cui et al., 2023)	2023	ADNI (442)	rs-fMRI	154	168/120^*db*^	–	ST-Aggregated Attention Network (–)	–	90.3^*cc*^	90.1^*ca*^	–	83.3^*ma*^
29	(Song et al., 2019)	2019	ADNI (48)	DTI	12	12/12^*db*^	12	Spectral-GCN (–)	–	–	–	–	89^*mb*^
30	(Guo et al., 2019)	2019	ADNI (327)	PET_AV45_	100	131/96^*db*^	–	Spectral-ChebNet (hierarchical SAG pooling)	–	–	93.0^*ce*^	–	77^*ma*^
31	(Li et al., 2023c)	2023	ADNI (230)	PET_AV45, AV1451_	113	117	–	Spectral-other (–)	–	–	93.5	–	–
32	(Cai et al., 2023)	2023	ADNI (839)	PET_AV45_	284	329/226^*db*^	–	Spectral-Other (–)	–	69.5^*cc*^	64.7^*ca*^, 75.1^*cb*^	–	–
			ADNI (1064)	PET_FDG_	335	330/399^*db*^	–		–	67.5^*cc*^	63.9^*ca*^, 67.0^*cb*^	–	–
33	(Klepl et al., 2022)	2022	in-house (40)	EEG	20	–	20	Spectral-GCN (Global maximum pooling)	91.9	–	–	–	–
34	(Shan et al., 2022)	2022	in-house (39)	EEG	20	–	19	ST-CNN (–)	91.1	–	–	–	–

^***da***^**MCI**: sMCI/pMCI; ^***db***^**MCI**: EMCI/LMCI; ^***dc***^**MCI**: EMCI; ^***dm***^**AD**: AD/MCI/ASD/ADHD/VCI; ^***ca***^NC/EMCI; ^***cb***^NC/LMCI; ^***cc***^EMCI/LMCI; ^***cd***^EMCI/AD; ^***ce***^LMCI/AD; ^***ba***^Balanced Accuracy.

Multilevel classification accuracy column reporting NC/MCI/AD classification or indicated separately, where ^***ma***^NC/EMCI/LMCI; ^***mb***^NC/EMCI/LMCI/AD.

ABIDE, Autism Brain Imaging Data Exchange; AD, Alzheimer's disease; ADHD, attention-deficit/hyperactivity disorder; ADNI, Alzheimer's Disease Neuroimaging Initiative; DTI, Diffusion Tensor Imaging; EEG, Electroencephalography; MCI, Mild Cognitive Impairment; EMCI, early-MCI; LMCI, late-MCI; sMCI, stable MCI; pMCI, progressive MCI; MRI, Magnetic Resonance Imaging; MRI, structural MRI; fMRI, functional MRI; rs-fMRI, resting state fMRI; VBM, Voxel-based morphometry; n, participant count; NC, Normal Control; NIFD, Frontotemporal lobar Degeneration Neuroimaging Initiative; OASIS, Open Access Series of Imaging Studies; PET, Positron Emission Tomography; PET(FDG), Fluorodeoxyglucose PET; PET(AV45), Amyloid PET; PET(AV1451), Tau PET; SMC, Significant Memory Concern; ST, Spatial-Temporal; VCI, Vascular Cognitive Impairments.

**Table 2:** Summary of key findings of the papers using GNNs and multimodal biomarkers for AD diagnosis.

**Sr**.	**Article**	**Year**	**Database (size)**	**Modalities**	**Subjects**			**GNN architecture**	**Classification accuracy (%)**				
					***NC***	***MCI***	***AD***	***FE-graph convolution (pooling)***	***NC/AD***	***sMCI/pMCI***	***NC/MCI***	***MCI/AD***	***Multilevel***
1	(Parisot et al., 2018)	2018	ADNI (540)	MRI+phenotypic	–	251	289	Spectral-ChebNet (–)	–	–	–	80	–
2	(Yang et al., 2023)	2023	TADPOLE (603)	MRI+(sex)	211	320	72	Spatial-other (global adaptive pooling)	97.1	–	97.2	92.4	–
3	(Song et al., 2021)	2021	TADPOLE (551)	MRI+(apoe4,age,sex)	–	315/236^*da*^	–	Spatial-GraphSAGE (–)	–	86.27	–	–	–
			TADPOLE (1,615)		413	865	337		–	–	–	–	94.06
4	(Kim, 2023)	2023	ADNI (506)	MRI+(apoe4, age, sex, edu)	214	292	–	Spectral-GCN (–)	–	–	–	–	–
5	(Li et al., 2023b)	2023	ADNI (502)	MRI+MO	161	133/75^*da*^	133	Spatial-Other (–)	88	71.1	87.21	–	–
6	(Yu et al., 2019)	2019	ADNI (126)	rs-fMRI+(age,sex)	44	44/38^*db*^	–	Spectral-GCN (Global Summation Pooling)	–	79.27^*cc*^	87.50^*ca*^, 89.02^*cb*^	–	–
7	(Jiang et al., 2020)	2020	ADNI (133)	rs-fMRI+(age, sex, site)	–	99	34	Spectral-GCN (Hierarchical Eigen Pooling)	–	–	–	78.5	–
8	(Zhu et al., 2021)	2021	ADNI (291)	fMRI+(age, sex)	94	86/67/44^*df*^	–	Spectral-ChebNet (Global Adaptive Pooling)	–	83.79^*cc*^	80.0^*ca*^, 80.77^*cb*^, 86.15^*cf*^, 88.18^*cg*^	–	–
9	(Li L. et al., 2022)	2022	ADNI (133)	fMRI+(sex, etc.)	–	99	34	ST-RNN (Hierarchical pooling)	–	–	–	86.7	–
10	(Bi et al., 2023)	2023	ADNI (870)	fMRI+(genetic)	237	197/203^*db*^	233	Spectral-other (–)	93.61	90.0^*cc*^	–	91.95^*ce*^	–
11	(Salim and Hamza, 2024)	2024	ADNI (573)	rs-fMRI+(age, sex, acquisition site)	402	171	–	Spectral-other (–)	–	–	98.25	–	–
12	(Kazi et al., 2019b)	2019	TADPOLE (564)	PET+(CSF, etc.)	160	320	84	Spectral-ChebNet (global summation pooling)	–	–	–	83.33	–
13	(Kazi et al., 2019a)	2019	TADPOLE (557)	PET+(CSF, etc.)	–	–	–	Spectral-ChebNet (global maximum pooling)	–	–	84.35	–	–
14	(Subaramya et al., 2022)	2022	ADNI (162)	MRI+DTI	100	–	62	Spectral-GCN (Global maximum pooling)	97	–	–	–	–
15	(Lin et al., 2023)	2023	ADNI (288)	MRI+PET	–	–	–	Spectral-GCN (–)	95.86	–	89.47	87.92	–
16	(Yang et al., 2022)	2022	ADNI (114)	fMRI+DTI	51	63	–	ST-RNN (global pooling)	–	–	90.4	–	–
17	(Meng and Zhang, 2023)	2023	ADNI (118)	fMRI+DTI	37	81	–	Spectral-other (global adaptive pooling)	–	–	90.7	–	–
18	(Lei et al., 2023)	2023	ADNI (184)	rs-fMRI+DTI	67	77/40^*db*^	–	MultiGraph-GCN (–)	–	92.31^*cc*^	85.42^*ca*^, 93.46^*cb*^	–	83.11^*ma*^
19	(Choi et al., 2022)	2022	ADNI (401)	DTI+PET_FDG+AV45_	89	132/55/53^*df*^	72	Spatial GAT (Global Adaptive Pooling)	–	–	–	–	96^*md*^
20	(Huang and Chung, 2020)	2020	TADPOLE (557)	MRI+PET_FDG_ + (apoe4, Phenotypic)	–	–	–	Spectral-ChebNet (–)	–	–	–	–	87.8
21	(Zhang et al., 2023e)	2023	ADNI (792)	MRI+PET_FDG_ + sex	246	120/211^*a*^	215	Spectral-ChebNet (–)	96.68	78	–	–	–
22	(Li W. et al., 2022)	2022	ADNI (502)	MRI+DTI+PET_AV45_	168	165	169	Spectral-Other (–)	96.06	–	92.73	95.15	90
23	(Zheng et al., 2022)	2022	TADPOLE (603)	MRI, PET, cognitive scores, CSF, risk factors, demographic	211	211/275^*da*^	72	Spatial-GraphSAGE (–)	–	92.3	–	–	92.31
24	(Li et al., 2023a)	2023	TADPOLE (564)	MRI+PET+(cognitive scores, CSF, risk factors, demographic)	–	–	–	Spectral-GCN (–)	99.3	88.3	98	94.6	–
25	(McCombe et al., 2022)	2022	ADNI (559)	MRI+PET_AV1451_ + cognitive scores	363	137	59	Spectral-GCN (–)	–	–	–	–	93.6^*ba*^
26	(Song et al., 2022)	2022	ADNI-2 (90)	fMRI+DTI+	29	34/27^*db*^	–	Spectral-GCN (Hierarchical TopK Pooling)	–	92.46^*cc*^	91.16^*ca*^, 94.22^*cb*^, 91.53^*cf*^, 95.71^*cg*^, 92.23^*ch*^	–	–
			ADNI-3, 200)	(sex, device, site)	64	52/40/44^*df*^							
			in-house (169)		70	99^*de*^							
27	(Xing et al., 2019)	2019	ADNI (368)	MRI+fMRI+(age, sex)	177	191^*dc*^	–	ST-RNN (–)	–	–	79.73^*ca*^	–	–
28	(Liu J. et al., 2020)	2020	ADNI (210)	MRI+rs-fMRI+(age, sex, MMSE)	105	105^*dc*^	–	Spectral-GCN (–)	–	–	84.1^*ca*^	–	–
29	(Chhabra et al., 2023)	2023	In-house	MRI+fMRI+DTI	600	–	600	ST-Other (–)	93.5	–	–	–	–
30	(Tian et al., 2023)	2023	ADNI (172)	MRI+fMRI+	65	65	42	Spatial-GraphSAGE (Hierarchical TopK Pooling)	88.71	–	79.68	82.71	–
			In-house (346)	DTI+(age, sex)	102	95	49						
31	(Qu et al., 2023)	2023	ADNI (318)	UNB, age, sex, cognitive scores	132	102	84	Spectral-GCN (–)	94.84	–	–	84.61	–
32	(Guo et al., 2023)	2023	ADNI (204)	Neuroimaging+(clinical, biological, genetic)	113	91	–	MultiGraph-GCN (–)	–	–	84.8	–	–
33	(Tekkesinoglu and Pudas, 2024)	2024	ADNI (2212)	Multimodal tabular data	754	1095	363	Spectral-GCN (–)	–	–	–	–	80.18
34	(Chen et al., 2024)	2024	ADNI (332)	MRI+PET_FDG(Synthesized)_	–	–	–	Spatial-GraphSAGE (–)	98.72	–	95.83	89.96	90.18^*nc*^, 83.08^*gc*^
													82.77^*mc*/*nc*^
35	(Zhang et al., 2022)	2022	ADNI (134)	MRI+fMRI+PET + (genetic, phenotypic)	100	–	34	Spectral-other (Global pooling)	–	–	–	82.09	–
					100	121	–		–	–	76.48	–	–
			ADNI (121)	–	–	80/41^*da*^	–		–	79.23	–	–	–
36	(Zhou et al., 2022a)	2022	ADNI (755)	MRI_VBM_	182	476	97	Spectral-GCN (Global Summation Pooling)	–	–	–	–	77.3
				MRI_VBM_+PET_FDG_					–	–	–	–	80.6
				MRI_VBM_+PET_FDG+AV45_					–	–	–	–	81.8
37	(Zhou et al., 2022b)	2022	ADNI (755)	MRI_VBM_	182	476	97	Spectral-GCN (–)	77.2^*aa*^	–	–	–	–
				MRI_VBM_+PET_FDG_					79.7^*aa*^	–	–	–	–
				MRI_VBM_+PET_FDG+AV45_					82.6^*aa*^	–	–	–	–
38	(Zhang et al., 2023d)	2023	ADNI (618)	MRI	172	186/103^*da*^	157	Spectral-ChebNet (–)	90.21	73.18	–	–	–
				PET					91.51	75.47	–	–	–
				MRI+PET					93.88	77.39	–	–	–
				MRI+PET+sex					94.8	79.24	–	–	–
				MRI+PET+apoe4					94.9	80.03	–	–	–
				MRI+PET+MMSE					96.94	79.24	–	–	–
39	(Zhang et al., 2023f)	2023	ADNI (898)	MRI	286	243/132^*da*^	237		88.95	75.64	–	–	–
				MRI+sex					90.08	76.37	–	–	–
			ADNI (792)	PET_FDG_	246	211/120^*da*^	215	Spectral-GCN (–)	87.43	73.2	–	–	–
				PET_FDG_+sex					88.86	75.2	–	–	–

^***da***^**MCI**: sMCI/pMCI; ^***db***^**MCI**: EMCI/LMCI; ^***dc***^**MCI**: EMCI; ^***dd***^**MCI**: SMC; ^***de***^**MCI**: LMCI; ^***df***^**MCI**: EMCI/LMCI/SMC.

^***ca***^NC/EMCI; ^***cb***^NC/LMCI; ^***cc***^EMCI/LMCI; ^***cd***^EMCI/AD; ^***ce***^LMCI/AD; ^***cf***^SMC/EMCI; ^***cg***^SMC/LMCI; ^***ch***^NC/SMC; ^***nc***^Node Classification; ^***gc***^Graph Classification; ^***ba***^Balanced Accuracy; ^***aa***^**:** average classification performance on three groups, including NC/AD, NC/MCI, and MCI/AD.

Multilevel classification accuracy column reporting NC/MCI/AD classification or indicated separately, where ^***ma***^NC/EMCI/LMCI; ^***mc***^NC/sMCI/pMCI; ^***md***^NC/EMCI/LMCI/SMC/AD.

AD, Alzheimer's disease; ADNI, Alzheimer's Disease Neuroimaging Initiative; APOE, apolipoprotein E; CSF, cerebrospinal fluid; DTI, Diffusion Tensor Imaging; EEG, Electroencephalography; GM, Gray Matter; MCI, Mild Cognitive Impairment; EMCI, early MCI; LMCI, late MCI; sMCI, stable MCI; pMCI, progressive MCI; MRI, Magnetic Resonance Imaging; MO, Morphological features (e.g., GM volume, Cortical thickness); MRI, structural MRI; fMRI, functional MRI; rs-fMRI, resting state fMRI; n, participant count; NC, Normal Control; PET, Positron Emission Tomography; PET(FDG), Fluorodeoxyglucose PET; PET(AV45), Amyloid PET; PET(AV1451), Tau PET; SMC, Significant Memory Concern; UNB, univariate neurodegeneration biomarker; VBM, Voxel-based morphometry; VCI, Vascular Cognitive Impairments.

## 3 Graph neural networks

This section provides a comprehensive overview of the fundamental background of GNNs, including their key components, framework, and taxonomy.

### 3.1 Overview

**Figure 3:**
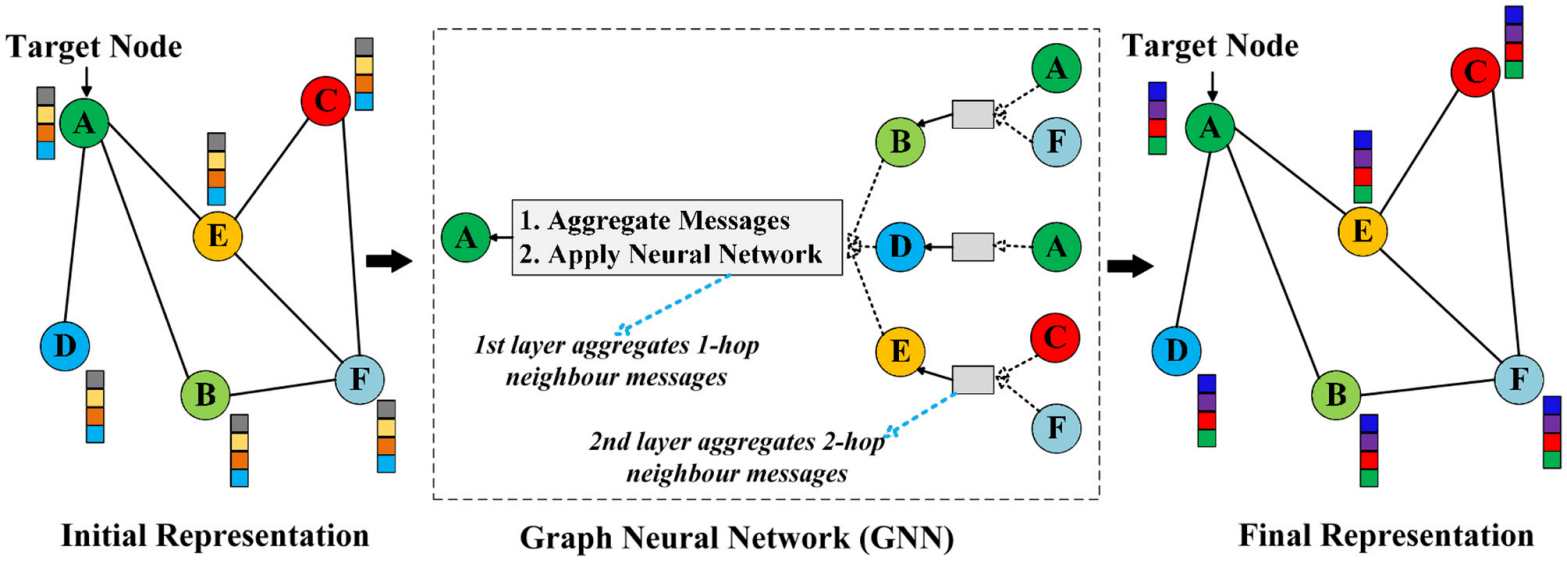
A high-level graphical illustration of how GNNs aggregate information from neighboring nodes to update node representations. For instance, it illustrates how a single node (i.e., A) in GNN aggregates messages from its local neighborhood. The GNN model aggregates messages from the local neighbors of node A (i.e., B, D, E). Subsequently, the messages from these nodes are derived from information aggregated from their respective neighborhoods, and so on. This illustrates a two-layer version of message-passing in a GNN model.

GNNs are a sophisticated branch of artificial neural networks specifically designed to handle data organized in graph structures. A graph is a mathematical representation of pairwise associations between entities, comprising a set of nodes *V* and edges *E*, denoted mathematically as *G* = (*V, E*). Nodes represent entities, while edges signify the relationships between them. The foundational concepts behind GNNs were inspired by early research that applied neural networks to directed acyclic graphs (Sperduti and Starita, 1997). Building on this, Gori et al. (2005) formally introduced GNNs, emphasizing the natural graphical representation of information. They argued for the necessity of models capable of processing graph-structured data directly. Subsequent studies by Scarselli et al. (2008); Gallicchio and Micheli (2010) demonstrated that GNNs could achieve significantly better performances than traditional ML and DL methods by iteratively leveraging graph topological information. These investigations are classified as recurrent GNNs (R-GNNs), which propagate neighbor information iteratively until achieving a stable representation of a target node. However, the computational cost associated with this process is substantial, leading to ongoing efforts to mitigate these challenges (Li et al., 2015; Dai et al., 2018). A defining characteristic of GNNs is their capability to perform operations on non-Euclidean data, which is particularly beneficial for tasks involving intricate relational structures. In contrast, traditional ML and DL methods are primarily designed for Euclidean data formats, such as images or sequential text, making them less effective for graph-structured data. GNNs address this limitation by employing local message aggregation and propagation across edges, enabling nodes to systematically gather information from their neighbors and refine their representations (Zhou et al., 2020). This ability to capture both local structures and global features allows GNNs to learn effectively from graph-structured data. Furthermore, GNN methods generate vector representations that encapsulate network topology and node features (Li M. et al., 2021), enhancing their effectiveness in processing graph-organized data. The high-level processes of message aggregation and propagation used to update node representations are illustrated in Figure 3.

Inspired by the success of Convolutional Neural Networks (CNNs) in computer vision, researchers have developed various approaches that redefine convolution for graph data. These methods are categorized as convolutional GNNs (ConvGNNs) or Graph Convolutional Networks (GCNs), which will be referenced later in this review. Following this trend, several variants of GNN architectures have emerged, including GCNs (Kipf and Welling, 2016), GraphSAGE (Hamilton et al., 2017), Graph Attention Networks (GATs) (Veličković et al., 2017), and Graph Isomorphism Networks (GINs) (Xu et al., 2018). Due to their ability to extract features based on data structure and automate feature extraction from raw inputs, GNN models have demonstrated exceptional performance across various domains (Liu and Zhou, 2022; Vashishth et al., 2020; Kwak et al., 2020).

### 3.2 GNN procedure in the context of AD diagnosis

**Figure 4:**
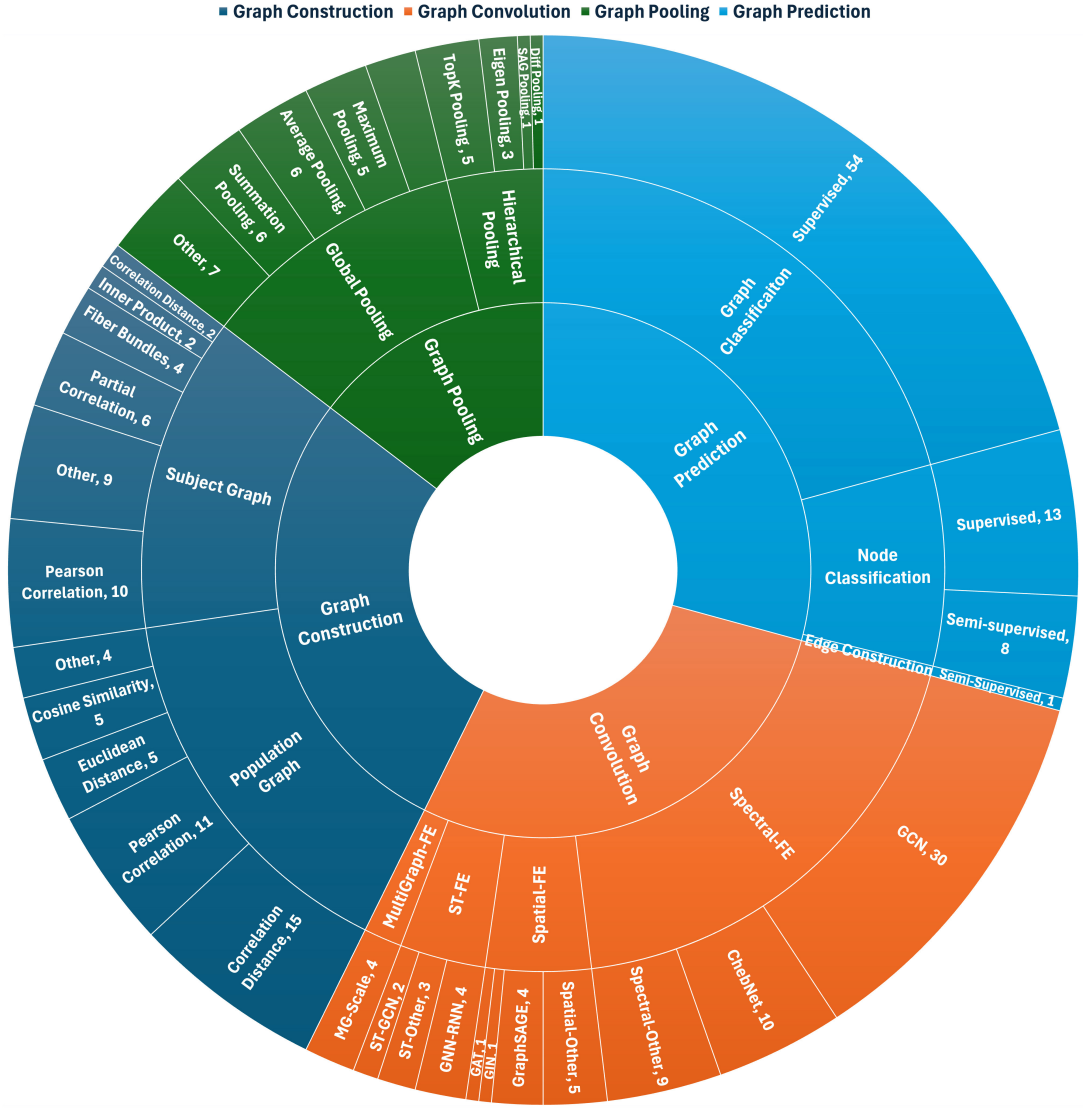
Sunburst plot depicting research trends for each GNN method in AD Studies. The plot is drawn from the works mentioned in Tables 1, 2. We note that most studies favored population graph construction methods using correlation distance and Pearson correlation, with the latter and WM fiber bundles common in individual graph construction. Spectral-FE methods predominated in feature extraction and graph convolution, while MultiGraph-FE was less frequent. ChebNet and GCN were the most used graph convolution methods. Hierarchical pooling was less common than global pooling, although TopK pooling was frequently reported within hierarchical approaches. Most studies focused on graph classification tasks with a supervised learning framework, and supervised node classification was more prevalent than semi-supervised or unsupervised methods. FE, Feature Extraction; MG, MultiGraph; ST, Spatial-Temporal; GAT, Graph Attention Network; GCN, Graph Convolutional Network; GIN, Graph Isomorphism Network; GNN, Graph Neural Network; RNN, Recurrent Neural Networks.

The GNN framework typically consists of four key computational modules: (i) graph construction, (ii) graph convolution, (iii) graph pooling, and (iv) graph prediction. The sunburst plot in Figure 4 illustrates the distribution of studies regarding GNN components and techniques within the context of AD diagnosis. The inner ring in the sunburst plot depicts the key GNN framework components, the middle ring illustrates the sub-methods or techniques associated with the key components of GNN, while the outermost ring represents the specific techniques used within each of the sub-methods or techniques.

#### 3.2.1 Graph construction

Graph construction is the initial phase of the GNN framework, involving the organization of data into a graph structure. Graphs can be categorized into two types: (i) individual (subject-level) graphs and (ii) population graphs. The following subsections categorize studies based on graph construction methods for both individual and population graphs.

**Individual graph:** Individual, or subject-level, graphs are typically constructed using predefined atlases or templates applied to neuroimaging scans. In these graphs, brain regions are represented as nodes, and the edges denote measures of structural, functional, or metabolic connectivity. Many studies (Ktena et al., 2018; Gu et al., 2021; Mei et al., 2022; Cui et al., 2023; Tian et al., 2023; Liu et al., 2023b; Wang Z. et al., 2023; Li et al., 2023c; Qin et al., 2022) have employed Pearson correlation to define connectivity, predominantly using fMRI data. Correlation distance has also been widely adopted as an alternative metric for edge construction, particularly in fMRI-based studies (Lee et al., 2021; Li X. et al., 2021). In contrast, another significant research focus involves building DTI-based connectivity networks utilizing fiber-tracking techniques (Choi et al., 2022; Subaramya et al., 2022; Chhabra et al., 2023), sometimes augmented with PET-derived information (Li W. et al., 2022). Several studies have also explored integrating multiple graph construction approaches (Yao et al., 2021; Zhou et al., 2022b; Liu et al., 2023c; Zhou et al., 2022a; Klepl et al., 2022; Fan et al., 2023). For example, Yao et al. (2021) employed templates of varying resolution and combined Pearson correlation with K-nearest neighbors (KNN), while Zhou et al. (2022b) calculated intra-subject connectivity using KNN. Liu et al. (2023c) proposed a two-phase strategy, constructing low- and high-order graphs with attention mechanisms, and Zhou et al. (2022a) integrated multimodal features into graph construction, with each node representing an ROI. Further, Klepl et al. (2022) applied multiple methods to derive functional connectivity networks from EEG data, and Fan et al. (2023) utilized an adaptive graph transformer to dynamically adjust the adjacency matrix based on extracted MRI features.

**Population graph:** Population graphs represent pairwise associations between subjects, where nodes correspond to individual subjects and edges reflect relationships based on demographic or feature similarities (Tekkesinoglu and Pudas, 2024; Salim and Hamza, 2024; Zhang et al., 2024). These graphs are constructed using both neuroimaging data (e.g., MRI, PET, fMRI) and non-imaging data (e.g., age, sex, genetic information), while connection-based statistics are commonly employed to quantify similarity between subjects (Salim and Hamza, 2024; Liu et al., 2023a; Ktena et al., 2018; Fan et al., 2022; Xing et al., 2019; Li L. et al., 2022; Zhang et al., 2024; Yu et al., 2019; Wen et al., 2022; Kim, 2023; Song et al., 2019; Zhao et al., 2019; Zhu et al., 2021; Parisot et al., 2018; Zhang et al., 2023f,d,e; Tekkesinoglu and Pudas, 2024; Li et al., 2023a,b; Wee et al., 2019; Yang et al., 2023; Meng and Zhang, 2023; Huang and Chung, 2020; Zheng et al., 2022; McCombe et al., 2022; Guo et al., 2023; Jiang et al., 2020; Song et al., 2022; Cai et al., 2023; Lei et al., 2023). Many studies leverage Pearson correlation to construct edges, particularly when using fMRI data and demographic variables (Salim and Hamza, 2024; Liu et al., 2023a; Ktena et al., 2018; Fan et al., 2022; Xing et al., 2019; Li L. et al., 2022; Zhang et al., 2024; Yu et al., 2019). For example, Li L. et al. (2022) applied thresholds to generate sparse brain networks, while Wen et al. (2022) integrated attention mechanisms to enhance graph construction. In fewer cases, structural connectivity graphs derived from DTI fiber bundles were aggregated into population graphs, with Pearson correlation used to determine inter-subject similarities (Song et al., 2019). Correlation distance is another common metric used to establish pairwise relationships (Zhao et al., 2019; Zhu et al., 2021; Parisot et al., 2018; Zhang et al., 2023f,d,e). For instance, Zhao et al. (2019); Zhu et al. (2021) developed functional connectivity networks for individual subjects using rs-fMRI, subsequently computing edge weights based on correlation distance and demographic features. Parisot et al. (2018) further integrated imaging and non-imaging data, using a Gaussian kernel to calculate adjacency matrices and combining them via the Hadamard product. Similarly, Zhang et al. (2023f,d) analyzed volumetric and metabolic features alongside phenotypic similarities, while (Zhang et al., 2023e) dynamically adjusted edge weights to reflect multimodal feature similarities. A smaller subset of studies utilized Euclidean distance to define edges within population graphs (Tekkesinoglu and Pudas, 2024; Li et al., 2023a,b; Wee et al., 2019; Yang et al., 2023). For example, Tekkesinoglu and Pudas (2024) computed it between cognitive scores to determine edge weights, and Li et al. (2023a) combined it with KNN for adjacency matrix construction. Cosine similarity has also been employed in some cases to quantify inter-subject associations (Meng and Zhang, 2023; Huang and Chung, 2020; Zheng et al., 2022; McCombe et al., 2022; Guo et al., 2023). Several studies combined multiple methods to optimize graph construction (Jiang et al., 2020; Song et al., 2022; Cai et al., 2023; Lei et al., 2023). For instance, Jiang et al. (2020) used functional connectivity derived from fMRI to inform edge weights, calculated via a Gaussian kernel. Song et al. (2022) developed a multi-center attention graph with attention mechanisms, while Cai et al. (2023) constructed a group-mean adjacency matrix incorporating second-order random walks. Additionally, Lei et al. (2023) created brain connectivity networks by combining local weighted clustering coefficients with structural and functional connectivity data from DTI and rs-fMRI, improving the representation of population graphs.

#### 3.2.2 Graph convolution

Following graph construction, graph convolution leverages the graph structure to enable message passing between nodes, facilitating the extraction of high-level features. We identified four fundamental techniques for feature extraction from neuroimaging data in GNN methods for AD diagnosis: (i) spectral feature extraction, (ii) spatial feature extraction, (iii) spatial-temporal feature extraction, and (iv) multigraph feature extraction.

**Spectral feature extraction (Spectral-FE):** Spectral feature extraction (Spectral-FE) operates in the spectral domain, treating the graph as a signal. It decomposes graph signals using the graph Laplacian eigenvalues, allowing convolution operations in the frequency domain via filtering. This approach provides a global perspective of the graph (Zhang et al., 2019). Notable Spectral-FE methods include ChebNet (Defferrard et al., 2016; Zhang et al., 2023e; Kazi et al., 2019a; Parisot et al., 2018; Liu et al., 2024; Ktena et al., 2018; Song et al., 2019), which utilizes Chebyshev polynomial approximations for graph convolution, and GCN, which, in constrast, simplifies Chebyshev convolution using a first-order approximation and is widely used in AD-related GNN methods (Kipf and Welling, 2016; Liu et al., 2023a; Wen et al., 2022; Jiang et al., 2020; Lee et al., 2021; Gu et al., 2021; Qin et al., 2022; Klepl et al., 2022). Recent studies have combined GCN and ChebNet (Zhao et al., 2019), while others (Li L. et al., 2022) employed GCN alongside spatial-temporal graph convolution, considering both structural and temporal features. Hybrid approaches include (Li et al., 2023c), which used GCN with a self-attention mechanism, and Fan et al. (2023), which combined GCN with ARMA layers for feature extraction. Studies like Meng and Zhang (2023) introduced multi-layer GCNs incorporating spectral-GCN and cluster-GCN to optimize efficiency. Other methods involved various convolution types, such as inter- and intra-community convolution (Bi et al., 2023) and feature concatenation from multiple filters (Yu et al., 2019).

**Spatial Feature Extraction (Spatial-FE):** In contrast to Spectral-FE, spatial feature extraction (Spatial-FE) directly applies convolution to nodes and their neighbors in the graph, similar to traditional image convolution, allowing for the extraction of spatial information between brain regions (Zheng et al., 2022). Spatial-FE methods are typically more scalable as they focus on local links rather than the entire graph structure. Key Spatial-FE techniques include GraphSAGE (Hamilton et al., 2017; Zheng et al., 2022; Chen et al., 2024), GAT (Veličković et al., 2017; Choi et al., 2022), and GIN (Xu et al., 2018; Wang Z. et al., 2023). GraphSAGE samples a fixed-size neighborhood and aggregates features using functions like mean or pooling, making it suitable for large graphs (Hamilton et al., 2017). In dynamic contexts, such as incorporating new subjects for diagnosis, traditional GNNs struggle with graph evolution. GraphSAGE addresses this by employing inductive learning through adjacent node sampling and aggregation, which is particularly useful in NDD diagnosis. For instance, Zheng et al. (2022) used GraphSAGE to partition population graphs into mini-batches, enabling inductive learning without requiring the entire graph. Spatial GraphSAGE was also employed for inductive representation learning in Chen et al. (2024). GAT enhances flexibility in capturing relationships through an attention mechanism that adapts edge weights during training, making it effective for brain connectivity analysis. Choi et al. (2022) combined GAT with heat kernel diffusion to control node neighborhood sizes adaptively. GINs, inspired by the Weisfeiler-Lehman test, utilize injective aggregation functions to match their power, with Wang Z. et al. (2023) applying GIN for spatial convolution to capture brain network structures and features, incorporating attention in the readout layer for node selection. Additionally, other approaches for spatial feature extraction include the spectral graph attention network (SpGat) (Xu et al., 2019) and bilinear aggregator (Zhu et al., 2020; Yang et al., 2023), as well as a model integrating CNN and GCN to extract local features and global connections (Fan et al., 2022).

**Spatial-temporal feature extraction (ST-FE):** Spatial-Temporal Feature Extraction (ST-FE) captures both spatial (node/edge interactions) and temporal (time-dependent) patterns, which are critical for dynamic graphs where nodes and edges evolve over time. This approach is especially relevant in brain networks derived from EEG and fMRI, where brain regions exhibit spatial correlations and generate temporal signals. To address these complexities, researchers have developed spatial-temporal graph convolution methods within GNN frameworks, integrating temporal dynamics into graph analysis (Yang et al., 2022; Kim et al., 2021; Chhabra et al., 2023). Prominent ST-FE techniques include GNNs combined with Recurrent Neural Networks (GNN-RNN) and Spatial-Temporal Graph Convolutional Networks (ST-GCNs). GNN-RNNs integrate GNNs with recurrent architectures such as Long Short-Term Memory (LSTM) networks or Gated Recurrent Units (GRUs) to capture spatial node dependencies alongside temporal dynamics. For instance, Yang et al. (2022) utilized a GNN-RNN model with GRU to aggregate multimodal brain network representations via spatial graph convolutions. Similarly, Xing et al. (2019) applied a sliding window approach to create dynamic functional networks, representing each network as a graph where MRI-derived node features were fed into an LSTM at each time step. Other studies leveraged GCN methods for spatial feature extraction across multiple time points, combining these results with LSTMs for temporal analysis (Li X. et al., 2021; Kim et al., 2021). In contrast, ST-GCNs jointly perform spatial and temporal convolutions to capture both static relationships among nodes and their dynamic progression over time (Zhang et al., 2019). Spatial convolutions are used to identify node associations, followed by temporal convolutions to model sequential patterns in node attributes. For example, Zhang et al. (2024) applied spectral-domain spatial graph convolutions alongside temporal convolutions to capture dynamic changes in functional connectivity and spatial correlations among brain regions. Shan et al. (2022) proposed an ST-GNN model comprising temporal convolution layers interleaved with spatial convolution layers, effectively modeling both dimensions. Hybrid approaches have also emerged, combining multiple techniques to enhance spatial-temporal modeling. For instance, Wang X. et al. (2023) employed spectral GCNs for feature aggregation, combined with GraphSAGE for neighborhood information, followed by spatial-temporal methods to analyze functional activity changes. Cui et al. (2023) introduced a dynamic graph attention mechanism to extract spatial-temporal features from fMRI time series data. Additionally, Chhabra et al. (2023) implemented a multimodal approach by applying CNNs for MRI, RNNs for fMRI, and GCNs for DTI, analyzing each modality independently before integrating them within a multimodal neural network for classification.

**MultiGraph feature extraction (MG-FE):** MultiGraph Feature Extraction (MG-FE) involves handling multigraphs, where multiple edges exist between the same nodes, each edge representing a unique relationship or interaction. Extracting features from multigraphs is challenging due to the need for multiple graph convolutions to capture diverse connections. MG-FE techniques are often classified by scale and construction method, with common scales and brain templates such as AAL116 (116 ROIs) (Liu et al., 2014) and CC200 (200 ROIs) (Wood et al., 2019). Construction methods like correlation distance and Pearson correlation (as discussed in Section 3.2.1) further differentiate these graphs. Several studies have applied MG-FE in multi-scale graph contexts. For example, Yao et al. (2021) utilized four brain templates, each generating a distinct graph and enabling high-order associations across subjects, while Yao et al. (2019) constructed multi-scale functional connections from three brain templates, aligning each template with a separate graph convolution branch. Similarly, Lei et al. (2023); Guo et al. (2023) used a multigraph approach to enhance feature extraction.

#### 3.2.3 Graph pooling

Following feature extraction through graph convolution, graph pooling is the next phase in GNNs, aiming to distill node embeddings into informative graph embeddings that highlight the most robust and distinctive features. Pooling is often synonymous with “graph readout” in the literature. Two main techniques are commonly used: global pooling and hierarchical pooling.

**Global pooling:** Global pooling methods convert node embeddings into graph-level embeddings, enabling a holistic representation of graph structures. Common approaches include adaptive, average, maximum, and summation pooling, each with distinct mechanisms and applications. Average Pooling computes the mean of node embeddings, capturing shared information across adjacent nodes. This technique has been widely used for dimensionality reduction and to represent both local and global brain structures. For instance, Qin et al. (2022); Kim et al. (2021) applied average pooling after graph convolutions to achieve compact graph-level representations, while studies like Jiang et al. (2020); Zhang et al. (2024) leveraged it to enhance cross-region classification tasks. Summation Pooling aggregates node embeddings by summing feature vectors, effectively creating a global representation of the graph. While this method efficiently combines features (Ktena et al., 2018; Fan et al., 2022; Zhou et al., 2022a), it may overlook relative feature importance. To address this, studies like Kazi et al. (2019b) employed weighted summation based on attention scores, allowing for a more nuanced integration of node features. Maximum Pooling emphasizes distinct features by selecting the maximum values from node embeddings. This approach is particularly useful for highlighting salient patterns in the data, as demonstrated in studies such as Klepl et al. (2022); Lee et al. (2021); Subaramya et al. (2022). Adaptive Pooling dynamically reduces the graph size while retaining structural information. Methods like those described in Choi et al. (2022); Meng and Zhang (2023); Zhu et al. (2021) employ custom layers to prioritize nodes based on their importance and connectivity, preserving key features during downsampling. For example, Zhu et al. (2021) implemented layers tailored to prioritize critical nodes, ensuring structural fidelity. Several studies have combined multiple pooling techniques to leverage their complementary strengths. For instance, Lin et al. (2023) integrated various pooling strategies, sometimes enhanced by additional mechanisms like attention modules (Wang Z. et al., 2023; Zhang et al., 2022), readout functions (Liu et al., 2023c), or GRU-based reasoning layers (Zhang et al., 2023a) to aggregate brain region-level features effectively.

**Hierarchical pooling:** While global pooling techniques can introduce noise from less relevant brain regions and may overlook community-level features, hierarchical pooling addresses these limitations by progressively reducing graph size layer by layer. This approach preserves community structures and characteristics, ultimately transforming node embeddings into graph-level representations. Key hierarchical pooling methods include TopK pooling, SAG pooling, Eigen pooling, and Diff pooling. TopK Pooling selects the top *K* nodes based on their importance, effectively coarsening the graph (Sebenius et al., 2021; Song et al., 2022; Tang et al., 2022; Tian et al., 2023). For example, Li X. et al. (2021) implemented a two-layer approach that reduced nodes by 50% at each layer, generating graph-level representations through mean and maximum pooling of the remaining nodes. SAG Pooling clusters nodes to maintain hierarchical graph structures during pooling (Guo et al., 2019), ensuring the preservation of important community-level features. Eigen Pooling utilizes eigenvectors of the graph Laplacian to summarize hierarchical node information. For instance, Jiang et al. (2020) incorporated Eigen pooling into a hierarchical population graph, while Wen et al. (2022) employed it to extract subgraph features prior to applying global average pooling for final graph-level representations. Diff Pooling reduces graph complexity by clustering nodes while retaining subnetwork properties. This method has been effectively used to preserve network integrity, as demonstrated in Mei et al. (2022). We note that several studies did not explicitly specify the pooling techniques used in their methodologies, highlighting a potential gap in the reporting of pooling strategies in the literature.

#### 3.2.4 Graph prediction

Graph prediction represents the final phase of the GNN framework, leveraging graph structures and node features to generate predictions at node, edge, or graph levels. Each prediction type aligns with distinct objectives (e.g., binary or multilevel classification; or MCI-to-AD conversion) and requires appropriate pooling, readout, or aggregation strategies.

**Node classification:** Node-level predictions involve classifying or regressing node labels, often within population graphs where nodes represent individual subjects. Supervised learning is the most popular framework for this task, with GNNs generating embeddings for AD prediction and disease progression analysis. For example, Liu et al. (2023a); Zhang et al. (2023e); Kim (2023) applied supervised GNNs to predict AD by embedding multimodal features, while Zhao et al. (2019) utilized a fully connected network with softmax layers for classifying early-MCI vs. late-MCI and NC vs. EMCI nodes. Multimodal approaches such as Peng et al. (2022) focused on integrating imaging and non-imaging features for disease classification. Semi-supervised learning has also been employed. For instance, Parisot et al. (2018) utilized semi-supervised GCNs to classify NC, MCI, and AD using imaging and demographic features. Similarly, Tekkesinoglu and Pudas (2024) integrated multimodal patient data for multiclass classification (NC, MCI, AD), achieving improved disease stratification. Other studies, including Salim and Hamza (2024); Tian et al. (2023); Qu et al. (2023), have extended semi-supervised learning techniques for node classification in the context of neurodegenerative diseases. A specialized node-level prediction task is disease progression analysis, focusing on the conversion of MCI to AD. Peng et al. (2022) introduced the FedNi framework, combining federated and graph learning to enhance model performance for this task. Additionally, Song et al. (2021) applied a metric-based meta-learning approach for early AD diagnosis using the TADPOLE dataset. Other significant contributions include attention-based models, such as Kazi et al. (2019b), which integrated LSTM mechanisms for multimodal feature learning, and interpretable GNN frameworks, such as Kim et al. (2021), which used longitudinal neuroimaging data to predict AD progression.

**Edge-level prediction:** Edge-level predictions, also referred to as link prediction, estimate the likelihood of edges forming between pairs of nodes. These tasks are particularly useful for inferring relationships or reconstructing incomplete graphs. For example, Peng et al. (2022) employed an unsupervised framework based on generative adversarial networks (GANs) to predict missing edges, helping to reconstruct brain connectivity patterns in graph-based models.

**Graph classification:** Graph-level prediction involves classifying entire graphs, where each graph typically represents an individual subject, often based on imaging or multimodal data. Pooling and readout operations are essential for transforming node-level embeddings into a compact graph-level representation. In supervised learning, Bi et al. (2023) classified subjects as NC or AD using spectral-based graph representations, while Huang and Chung (2020) employed MLPs with fusion layers to classify healthy vs. diseased subjects. Similarly, Shan et al. (2022) flattened node features after convolution and pooling, employing a fully connected layer for classification. A variety of graph classification techniques have been utilized to predict different AD stages. Multimodal frameworks integrating functional and structural data were developed by Zhang et al. (2023d); Zhou et al. (2022a); Meng and Zhang (2023), while Song et al. (2022) employed dynamic graph structures with attention mechanisms. Kazi et al. (2019b) enhanced interpretability by integrating graph attention networks (GATs) with LSTM-based reasoning. Furthermore, Wen et al. (2022); Tang et al. (2022); Zhu et al. (2021) utilized a combination of spectral and spatial graph convolutions to capture higher-order graph properties. Pooling and readout mechanisms have been a focus of several studies for improving graph-level predictions. Mei et al. (2022) employed Diff Pooling to preserve subnetwork features, while Jiang et al. (2020) utilized Eigen Pooling to retain hierarchical graph information. Numerous other works have contributed to advancing graph classification for AD stage prediction, including Gu et al. (2021); Cui et al. (2023); Liu et al. (2023b); Li et al. (2023c); Choi et al. (2022); Zhang et al. (2022); Lee et al. (2021); Yao et al. (2021); Lin et al. (2023); Aafiya and Jeyachidra (2024); Yang et al. (2023); Fan et al. (2022); Li L. et al. (2022); Song et al. (2019); Chhabra et al. (2023); Zhang et al. (2023f); Wang Z. et al. (2023); Li et al. (2023b); Cai et al. (2023); McCombe et al. (2022); Klepl et al. (2022); Zuo and Kamata (2023); Kazi et al. (2019a); Liu et al. (2023c); Yang et al. (2022); Wang X. et al. (2023); Fan et al. (2023); Guo et al. (2023); Zhang et al. (2024); Guo et al. (2019); Qin et al. (2022); Li et al. (2023a); Lei et al. (2023); Kim et al. (2021); Liu et al. (2024); Zhang et al. (2023a); Hao et al. (2024); Zhu et al. (2022a); Kumar et al. (2022); Liu X. et al. (2020), employing diverse techniques and frameworks to enhance prediction accuracy and robustness across AD stages. Additionally, fewer studies, such as Li L. et al. (2022); Zhu et al. (2021), reported the disease progression prediction at the graph level. Li L. et al. (2022) proposed an ensemble framework incorporating hierarchical GCN and transfer learning to improve the predictive performance for MCI to AD progression. Likewise, graph-based models like the structure and feature-based graph U-Net (SFG U-Net) (Zhu et al., 2021) demonstrated the utility of integrating high-order structural and node features for this purpose.

## 4 Results: GNN for AD diagnosis

This section surveys the application of GNNs in classifying subjects with normal cognition, mild cognitive impairment, and AD, focusing on both unimodal and multimodal approaches. We analyze the literature based on dataset size and modality, classification accuracy, and levels, including binary (e.g., NC/AD), multilevel classifications (e.g., NC/MCI/AD), and disease progression (e.g., MCI-to-AD conversion). Detailed findings on GNN-based AD diagnosis are presented in Tables 1, 2. Furthermore, we have listed the verified GitHub repositories for studies having open-source code in Supplementary Table S1 to facilitate replication and serve as a useful resource for researchers in the academic community. This table lists the study reference and related URL, providing a direct pathway for readers to dive into the source code.

### 4.1 Unimodal data

We first present GNN-based studies for AD diagnosis using unimodal neuroimaging data. Table 1 presents key insights from studies utilizing GNN methods alongside unimodal biomarkers for AD diagnosis.

**Magnetic resonance imaging (MRI):** GNNs have been widely applied to MRI data for AD diagnosis (Fan et al., 2023, 2022; Wee et al., 2019; Liu et al., 2024; Zhang et al., 2023a; Kim et al., 2021; Peng et al., 2022; Aafiya and Jeyachidra, 2024; Hao et al., 2024; Zhu et al., 2022b). For example, Zhang et al. (2023a) proposed a multi-relation reasoning network leveraging structural MRI to capture spatial and topological features, while Peng et al. (2022) introduced a federated learning framework combined with a graph GAN to handle missing data and train a global GCN node classifier for MCI and AD classification. Fan et al. (2023) developed a graph reasoning module using an adaptive graph transformer to generate graph representations from CNN feature maps, enhancing AD diagnostic accuracy. Similarly, Fan et al. (2022) presented BGL-Net, a global-local information fusion network integrating CNNs and GCNs for robust classification. Spectral graph CNNs incorporating cortical thickness and geometric parameters were applied by Wee et al. (2019) to detect MCI and AD. For longitudinal MRI, Hao et al. (2024) employed weighted hypergraph convolutional networks, while Liu et al. (2024) proposed a dual-structure hierarchical graph learning framework combining individual and population models.

**Functional MRI (fMRI):** fMRI has also been extensively analyzed with GNNs for AD diagnosis (Zhang et al., 2023e; Ktena et al., 2018; Zhao et al., 2019; Liu X. et al., 2020; Yao et al., 2021; Gu et al., 2021; Lee et al., 2021; Kumar et al., 2022; Wang X. et al., 2023; Tang et al., 2022; Mei et al., 2022; Wen et al., 2022; Qin et al., 2022; Liu et al., 2023b; Zuo and Kamata, 2023; Liu et al., 2023c; Wang Z. et al., 2023; Cui et al., 2023). Ktena et al. (2018) used multilayer GCNs to predict AD by assessing graph similarity, while Gu et al. (2021) developed a fully supervised GCN that performs automatic feature selection from brain connectivity networks for disease stage classification. A personalized dual-branch GNN with spatio-temporal attention was proposed by Cui et al. (2023) for MCI detection, and Wang Z. et al. (2023) combined dynamic multi-task GINs with attention mechanisms to improve AD classification while simultaneously predicting age and sex. Adaptive multi-view graph classifiers were applied by Liu et al. (2023b) to reduce overfitting, and Liu X. et al. (2020) employed a Siamese GCN for effective graph representation in MCI/AD classification. Approaches like Lee et al. (2021); Qin et al. (2022) introduced unified frameworks and U-shaped hierarchical GCNs for disease detection. Most research in brain dynamics has relied on static functional brain networks, which fail to capture temporal variations in brain activity. Studies such as Wang X. et al. (2023); Tang et al. (2022); Mei et al. (2022) advocate for dynamic functional networks for a more accurate understanding of brain signal variations. Yao et al. (2021) developed a mutual multiscale triplet GCN that constructs a coarse-to-fine brain structural network using multiple parcellation templates. Similarly, Liu et al. (2023a) proposed a multiscale-atlases-based hierarchical GCN for analyzing functional connectivity networks. Wen et al. (2022) introduced a multi-view GCN (MVS-GCN) that combines graph structure learning with multi-task graph embedding to improve classification in AD diagnosis. Lastly, (Zuo and Kamata 2023) utilized a hypergraph convolutional network with attention mechanisms focused on the default mode network (DMN) to enhance classification performance.

**Positron emission tomography:** Some studies have applied PET data in conjunction with GNN methods for AD diagnosis (Cai et al., 2023; Li et al., 2023c; Guo et al., 2019). For example, Cai et al. (2023) introduced a brain network-specific hypergraph neural network to analyze the propagation of neuropathological events in AD. Li et al. (2023c) developed the multiple protein features network (MPN) and higher-order MPN to enhance MCI detection using PET scans. Additionally, Guo et al. (2019) proposed PETNet, a generalized graph-based CNN architecture for 3D PET image classification.

**Electroencephalography:** Fewer studies employed EEG data for diagnosing AD. Klepl et al. (2022) utilized a GNN-based framework to classify AD patients using sensor-level EEG signals, incorporating eight functional connectivity measures to estimate EEG brain graphs. Shan et al. (2022) developed a dynamic spatio-temporal GCN for early AD diagnosis using EEG data.

**Diffusion tensor imaging:** Graph-based methods for DTI have been less explored. Song et al. (2019) developed a multiclass GCN classifier based on structural connectivity, outperforming SVMs for AD stage classification.

### 4.2 Multimodal data

The key findings of the studies utilizing multimodal data for AD diagnosis combining imaging (e.g., MRI, PET, DTI, fMRI) and non-imaging data (e.g., age, sex, education, CSF, APOE4, genetic factors) are summarized in Table 2.

Several studies focused on integrating single-modality imaging and phenotypic data types for AD diagnosis, primarily through GCNs (Parisot et al., 2018; Yang et al., 2023; Kim, 2023; Li et al., 2023b; Song et al., 2021; Yu et al., 2019; Salim and Hamza, 2024; Jiang et al., 2020). For instance, Parisot et al. (2018); Yang et al. (2023); Kim (2023); Li et al. (2023b); Song et al. (2021) developed GCN frameworks that combined MRI and phenotypic data, representing subjects as a sparse graph to enhance AD diagnosis. (Yu et al., 2019) proposed a multiscale GCN for the diagnosis of MCI from rs-fMRI and phenotypic data. The aggregator normalization GCN introduced by Salim and Hamza (2024) improved predictive capabilities through the integration of diverse features, while Jiang et al. (2020) developed a hierarchical GCN designed to enhance graph embedding learning by merging global population networks with individual brain networks. Several other studies incorporated multimodal neuroimaging data into GNN frameworks for AD diagnosis (Subaramya et al., 2022; Lin et al., 2023; Yang et al., 2022). For example, Subaramya et al. (2022) combined MRI and DTI scans to construct structural brain graphs for classification tasks. Similarly, Lin et al. (2023) merged MRI and PET features using GCNs to boost classification performance. The multimodal dynamic GCN proposed by Yang et al. (2022) focused on learning structural and functional network features from fMRI and DTI data. Additionally, studies such as Zhang et al. (2023e); Huang and Chung (2020); Zhang et al. (2023d) successfully combined multimodal neuroimaging and non-imaging data within GNN methods. Huang and Chung (2020) proposed an uncertainty-aware disease prediction framework combining multimodal imaging and non-imaging data. Furthermore, Zhang et al. (2023d) developed a joint CNN-GNN framework that extracts imaging features via CNN and integrates these with non-imaging data through GNNs.

This subsection provides a comparative overview of recent approaches to Alzheimer's Disease (AD) diagnosis. Specifically, it contrasts unimodal and multimodal strategies, examines accuracy trends across datasets and populations, and evaluates methodological differences in GNN-based architectures. This subsection presents a comparative analysis of recent methodologies for diagnosing Alzheimer's Disease (AD). This study contrasts unimodal and multimodal strategies, examines accuracy trends across datasets and populations, and evaluates methodological differences in GNN-based architectures.

### 4.3 Comparative perspectives on AD diagnosis with GNNs

This subsection presents a comparative analysis of recent approaches for diagnosing AD, based on the studies reviewed in Sections 4.1, 4.2. This study contrasts unimodal and multimodal strategies, examines accuracy trends across datasets and populations, and evaluates methodological differences in GNN-based architectures.

#### 4.3.1 Unimodal vs. multimodal approaches

**Figure 5:**
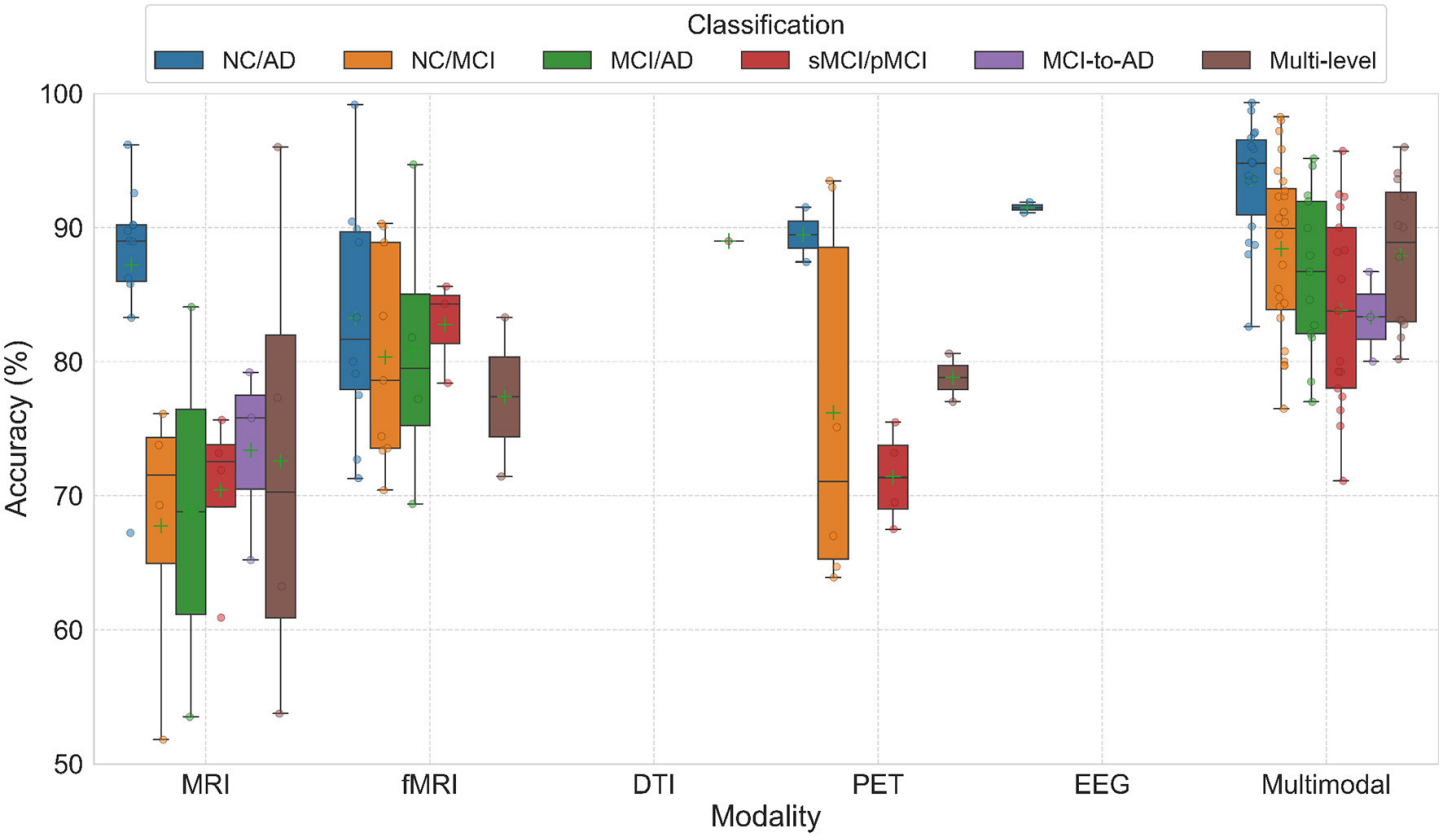
The boxplots display the accuracy of AD diagnosis across unimodal and multimodal data approaches. Multimodal methods were the most commonly reported in the reviewed studies. Among unimodal approaches, fMRI was the most frequently utilized. Mean accuracy values are marked with plus symbols, and individual data points are represented by circles. The boxplots are based on classification accuracies reported in the studies listed in Tables 1, 2.

In AD diagnosis studies, the difference between unimodal and multimodal techniques is crucial. We conducted a comparative analysis to evaluate the effectiveness of unimodal versus multimodal approaches for AD diagnosis. The results shown in Figure 5 demonstrate that integrating multimodal neuroimaging data consistently yields superior classification performance compared to single-modality methods. We note that in total, among the 73 reviewed studies, 39 utilized multimodal data, while 34 employed unimodal data.

**Figure 6:**
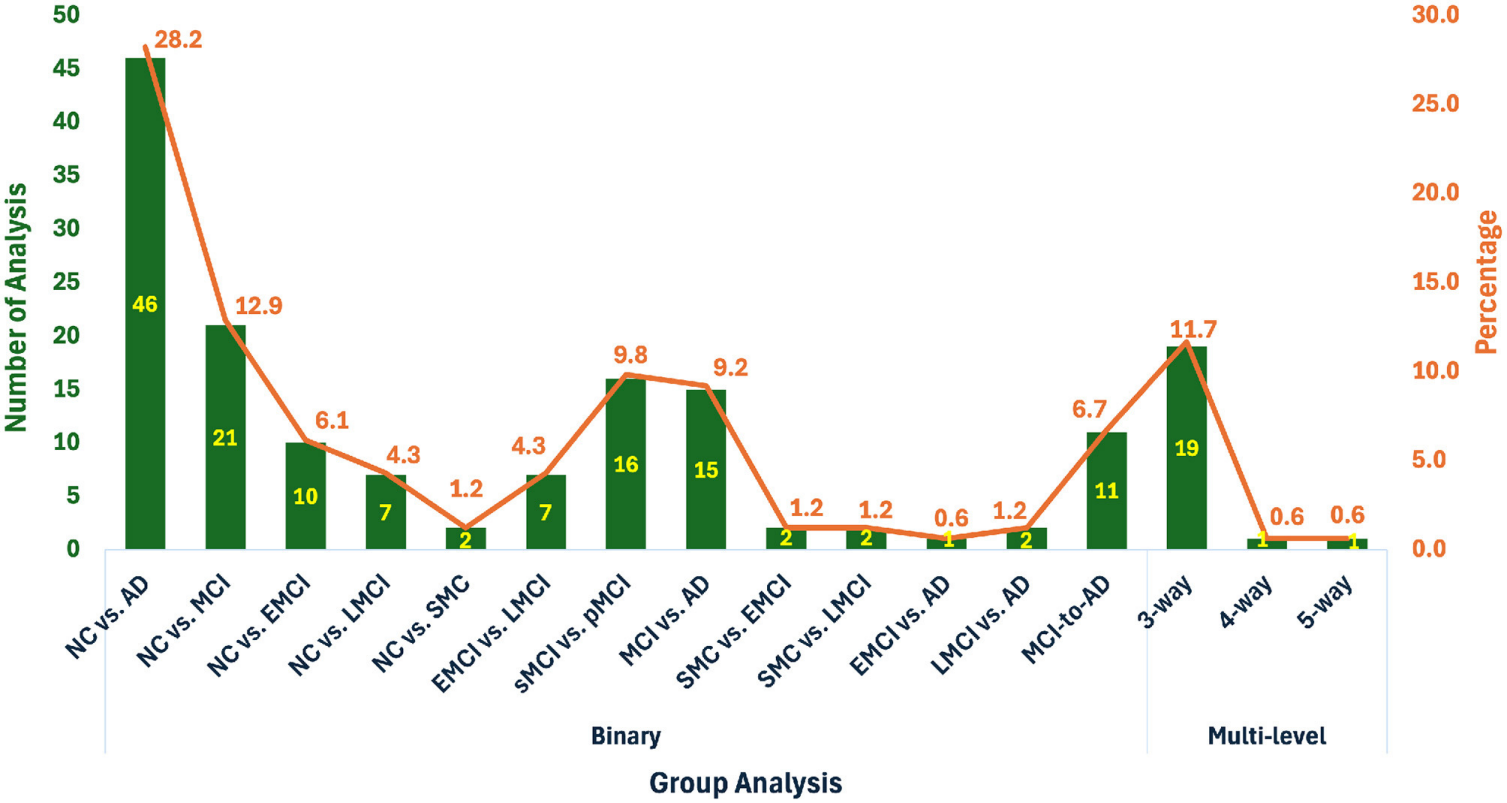
Frequency and percentage of classification task for each group of analysis in the reviewed studies. The plot is drawn from the studies mentioned in Tables 1, 2.

Unimodal methods predominantly employed fMRI, achieving high accuracy across binary classification tasks (e.g., NC/AD), with accuracies ranging from 71.3% to 99.16%. Structural MRI studies also demonstrated good performance, though they were generally less effective than fMRI, particularly for differentiating NC/MCI/AD. PET studies showed mixed performance, with better results observed in NC/AD tasks, though their overall use was limited. Additionally, EEG was applied in only two studies, and DTI was used in just one. Multimodal strategies demonstrated substantial improvements in classification accuracy compared to unimodal methods. For instance, leveraging TADPOLE data, which integrated imaging (MRI, PET) and non-imaging variables (cognitive tests, CSF, risk factors), achieved a maximum accuracy of 99.3% for the NC/AD classification task. Other tasks, such as NC/MCI and MCI/AD, also exhibited strong results, with top accuracies of 98.25% and 95.15%, respectively. Figure 6 highlights the distribution of classification tasks across the studies. Binary classification tasks, particularly NC/AD, were most common, followed by NC/MCI. Multilevel classification (e.g., NC/MCI/AD) and progression analysis (MCI-to-AD conversion) were less frequently addressed, with the latter representing only 11% of tasks.

#### 4.3.2 Accuracy trends across datasets and populations

**Figure 7:**
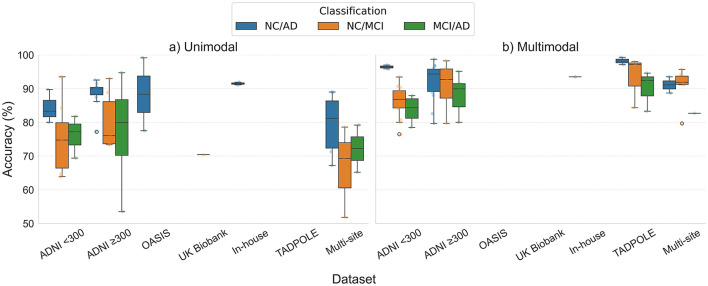
The boxplots represent the distribution of reported GNN classification accuracies for AD diagnosis across different datasets and population size, stratified by **(a)** unimodal and **(b)** multimodal approaches. The results are shown for NC vs. AD, NC vs. MCI (where NC could be NC or SMC; MCI could be EMCI, LMCI), and MCI vs. AD (where MCI could be EMCI, LMCI) classification tasks. The plot is drawn from the studies mentioned in Tables 1, 2.

Diagnostic accuracy varies among datasets and populations, underscoring the impact of sample composition, demographic diversity, and data quality. This subsection presents the performance of GNNs across different datasets and population sizes. We conducted a comparative analysis to evaluate the generalizability and robustness of GNN methods across different datasets, such as ADNI, OASIS, TADPOLE, UK Biobank, and in-house etc. The findings highlighted that ADNI is the most widely used dataset (unimodal: 24/34; multimodal: 29/39) among all other datasets used in reviewed studies, followed by multi-site (unimodal: 4/34) and TADPOLE (multimodal: 7/39). The results are summarized in Figure 7. The results indicated that overall multimodal studies consistently reported higher median accuracies compared to unimodal approaches. This effect was particularly evident in larger and harmonized datasets like ADNI and TADPOLE, where multimodal models showed superior median performance and lower variability, indicating greater robustness and generalizability. In contrast, unimodal studies exhibited greater variability in results, especially for complicated tasks such as NC vs. MCI and MCI vs. AD. NC/MCI classification, highlighting the limitations of single-modality inputs for early disease detection. Furthermore, NC vs. AD classification demonstrated the highest consistency and performance across all datasets, often surpassing 85%–90% accuracy. Conversely, NC vs. MCI and MCI vs. AD tasks exhibited reduced and more variable accuracies, especially in unimodal studies. Multimodal approaches have been shown to reduce this variability, indicating that the integration of structural, functional, and demographic features improves sensitivity to subtle disease-related patterns.

Furthermore, it is found that the population size varied significantly across studies conducting using ADNI dataset [unimodal: 48 (Song et al., 2019) – 1,644 (Zhang et al., 2023a); multimodal: 114 (Yang et al., 2022) – 870 (Bi et al., 2023)], therefore we further stratified the studies based on population size conducted using ADNI data (such as studies with sample size less than 300 subjects and more than 300 subjects). The findings highlighted the size of the population as a significant factor in the performance of GNN. It is evident that the studies conducted using ADNI ≥300 samples reported superior performance and less variable accuracies as compared to studies using sample sizes of less than 300, which underscores the impact of population size on the consistency and stability of GNNs.

Several studies (unimodal: Wee et al., 2019; Fan et al., 2022; Liu et al., 2023b,a; multimodal: studies Song et al., 2022; Tian et al., 2023) reported datasets from multi-site/multi-center (such as ANDI, OASIS, ABIDE, in-house, etc.). The findings revealed that studies conducted using multi-site datasets introduced increased variability, especially in unimodal studies, indicating heterogeneity across acquisition protocols. In contrast, large or harmonized datasets like ADNI ≥300 or TADPOLE yielded greater stability and accuracy, especially when combined with multimodal integration. In Summary, these findings emphasize that the dataset, population size, and modality integration are critical to enhance the generalizability and robustness of GNN-based approaches for AD diagnosis. Specifically, large and multimodal datasets enhance the reliability and generalizability of GNN models compared to small or unimodal datasets. Thus, by addressing the implications of data heterogeneity and domain shift, multimodal and large-scale datasets tend to offer the most reliable foundation for developing GNN-based methods for neurodegenerative diseases.

Methodological choices in graph neural network-based approaches, including graph construction strategies, aggregation functions, and learning objectives, directly influence diagnostic performance. This subsection presents a comparative analysis of various GNN architectures utilized in AD diagnosis, highlighting their respective advantages and trade-offs.

Methodological choice within GNN-based methods about graph construction methodologies, aggregation functions, and learning objectives have a direct impact on diagnostic performance in graph neural network-based approaches. This subsection presents a comparative analysis of various GNN architectures utilized in AD diagnosis, highlighting their respective advantages and trade-offs.

#### 4.3.3 Methodological comparison of GNN architectures for AD diagnosis

**Figure 8:**
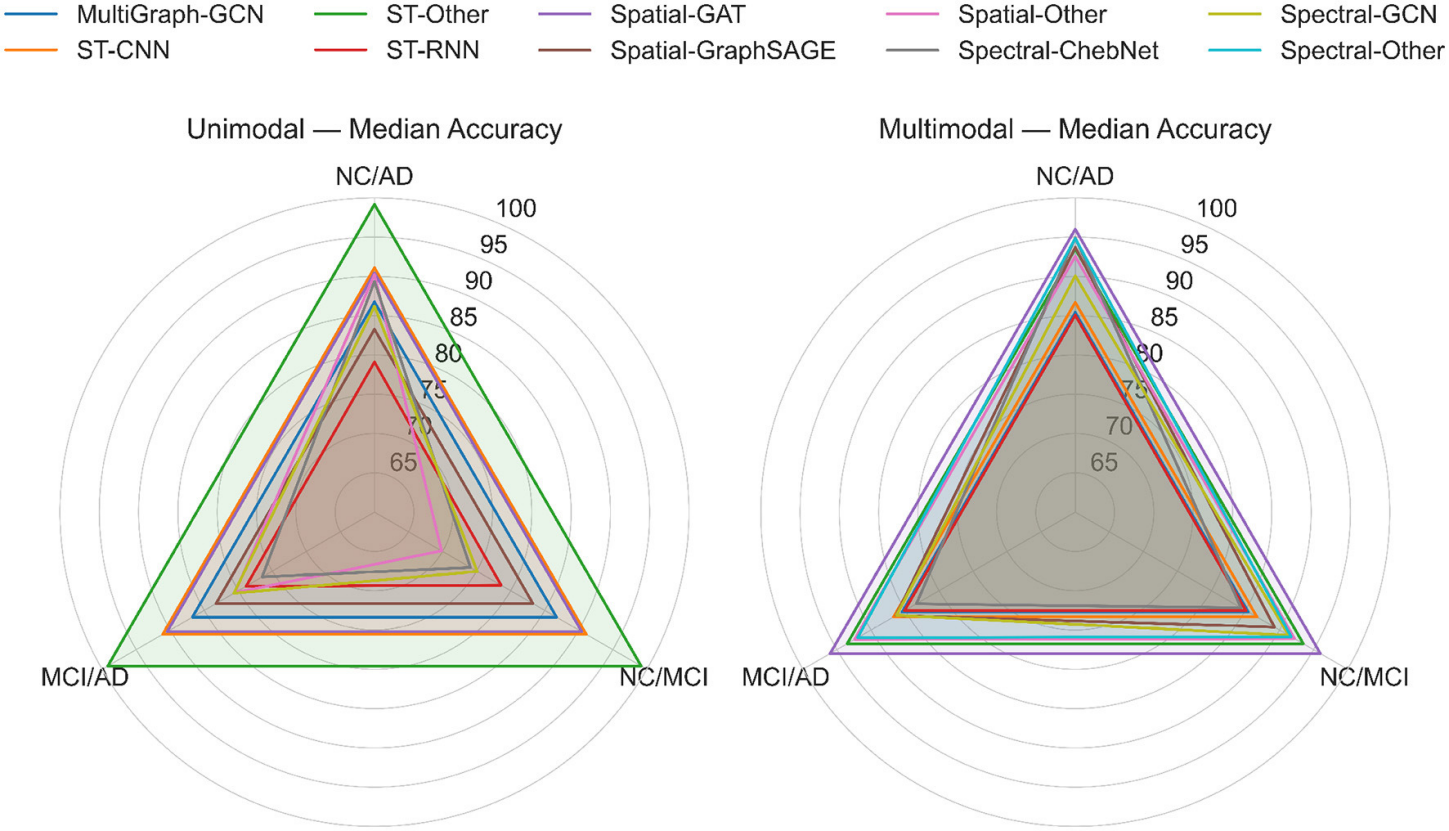
Radar plots highlight the median classification accuracies reported across families of GNN for AD diagnostic tasks (NC vs. AD, NC vs. MCI, and MCI vs. AD) in unimodal **(left)** and multimodal **(right)** studies. The rings in the radar plots represent accuracy thresholds ranging from 65% to 100%, whereas each polygon represents a distinct GNN family. The results are shown for NC vs. AD, NC vs. MCI (where NC could be NC or SMC; MCI could be EMCI, LMCI), and MCI vs. AD (where MCI could be EMCI, LMCI) classification tasks. The plot is drawn from the studies mentioned in Tables 1, 2, and analysis results in Supplementary Table S2.

Diagnostic performance in GNN-based approaches is directly impacted by methodological decisions, such as graph construction methods, graph convolution, etc. This subsection presents a critical analysis across families of GNN architectures for AD diagnosis and classification tasks, highlighting their respective advantages and trade-offs. Figure 8 highlights a comparative synthesis of GNN architectures using radar plots for both unimodal and multimodal studies, revealing the variations in stability and task sensitivity among families of GNNs. The distribution of studies and accuracies by GNN architecture and classification task is given Supplementary Table S2, where data derived from Tables 1, 2. The findings of the analysis indicated that spectral methods-such as GCN, ChebNet-are most prevalent both in the number of studies as well as their reliability across all AD diagnostic tasks. The spectral-GCN is the most widely used GNN architecture (unimodal: 18/34; multimodal: 14/39) (source: Tables 1, 2). The findings revealed that spectral GCN-based studies consistently reported accuracies over 90% in NC vs. AD classification task; however, exhibited reduced and more variable accuracies for MCI vs. AD [unimodal: 53.5% (Kim et al., 2021)–94.7% (Gu et al., 2021); multimodal: 78.5% (Jiang et al., 2020)–94.6% (Li et al., 2023a)]. This indicates that although spectral convolutions effectively identify significant global differences between NC and AD, they are less adept at recognizing more nuanced progression patterns. In contrast, ChebNet, despite its limited applications (unimodal: 5/34; multimodal: 7/39), consistently demonstrated high performance (unimodal: 85.8%–91.51%; multimodal: up to 96%–97%) for the NC vs. AD classification task as well as exhibits greater stability across classification tasks, indicating the effectiveness of polynomial filters for handling noise and sparse graphs.

In contrast to the spectral methods, spatial GNN methods-such as GraphSAGE, GAT, GIN-were not frequently reported in either unimodal or multimodal studies. Despite being less studied, spatial GNN architectures have several advantages. Spatial-GraphSAGE methods (unimodal: nan; multimodal: 4/39) indicated competitive and consistent performance across AD diagnostic classification tasks [NC vs AD: 98.72%; NC vs. MCI: 95.83%; MCI vs. AD: 89.96% (Chen et al., 2024)], which underscores the inferential significance of sampling-based aggregation in the context of multimodal inputs. However, GIN architecture was observed only once in an unimodal study, achieving 91.1% in NC vs. AD classification (Wang Z. et al., 2023). Likewise, GAT was observed in a single study (Choi et al., 2022). Their limited use in literature constrains conclusions; nonetheless, their expressiveness suggests opportunities for future research.

Similar to spectral GNN architectures, Spatio-temporal (ST) GNN architecture-RNN and CNN-based GNN have been investigated in both unimodal and multimodal settings; however, they are still underrepresented (unimodal: 3; multimodal: 4). In unimodal studies, ST-Other (which is a hybrid architecture integrating spectral GCNs for feature aggregation with GraphSAGE for neighborhood information, followed by ST methods to analyze functional activity changes) reported the highest accuracy (99.16%) in NC vs. AD classification task (Wang X. et al., 2023). On the other hand, ST GNN methods reported competitive performances across AD diagnostic tasks; however, these are not yet fully exploited by current implementations. In addition, MultiGraph GNN methods (unimodal: 2; multimodal: 2) reported accuracies ranging from 83.4% to 93.46%, indicating potential for subject-level heterogeneity. However, the limited number of studies in the literature impacts definitive conclusions. Overall, the methodological comparison of GNN architectures for AD diagnosis suggested that spectral methods (GCN, ChebNet) offered robust and stable baseline performance. Spatial methods, such as GraphSAGE and GAT, demonstrated potential in multimodal integration (e.g., MRI + PET + clinical features); however, the supporting evidence remains limited. Spatio-temporal methods are still in their early phase, exhibiting only limited advancements.

### 4.4 Explainability in GNNs

Explainability and interpretability of GNNs are critical for their clinical adoption. Interpretable models can pinpoint specific brain regions and neural connections driving predictions, providing insights into underlying pathologies and informing treatment strategies. Conversely, a lack of explainability reduces GNNs to black-box models, hindering their integration into medical decision-making, where transparency and accountability are essential (Li X. et al., 2021; Ying et al., 2019).

Various methods for interpreting GNN predictions have been reported in the literature (Zhou et al., 2022a,a; Selvaraju et al., 2017; Wang X. et al., 2023; Liu et al., 2023a; Kim et al., 2021; Ying et al., 2019; Kim, 2023; Zhang et al., 2022; Wee et al., 2019; Fan et al., 2022; Li X. et al., 2021; Cui et al., 2021; Lee et al., 2021; Gu et al., 2021). Zhou et al. (2022a) introduced an interpretable Gradient Class Activation Mapping (Grad-CAM) approach (Selvaraju et al., 2017; Zhang et al., 2024) to analyze key regions of interest. This framework highlighted the putamen and pallidum as critical biomarkers for distinguishing normal cognition, MCI, and AD groups, while also identifying discriminative features based on brain connectivity patterns. Similarly, Wang X. et al. (2023) employed Grad-CAM to identify important brain regions, revealing the hippocampus and temporal pole's significance in AD classification tasks. In addition, Liu et al. (2023a) used Grad-CAM to interpret GNN predictions by pinpointing significant brain regions derived from fully connected networks, aiding in the differentiation between disease stages. Zhang et al. (2024) combined Grad-CAM with spatio-temporal GCNs to assess the individual impact of each brain region on classification, producing heatmaps that emphasized key areas associated with disease progression. Furthermore, Kim et al. (2021) applied the GNNExplainer (Ying et al., 2019) to elucidate model predictions by identifying essential nodes and features in the graph, thus highlighting subgraph structures that contribute significantly to the model's outcomes. Similarly, (Kim 2023) utilized GNNExplainer to rank personalized risk factors for AD prediction, revealing unique biomarker patterns across different groups and underscoring variability in AD progression. Beyond Grad-CAM and GNNExplainer, other techniques for enhancing GNN explainability have emerged, including attention mechanisms, pooling scores, leave-one-region-out methods, and connectograms. For example, Zhang et al. (2022) developed a local-to-global GNN that integrates individual-level functional connections with population-level non-imaging data, successfully capturing both local and global features based on self-attention scores. Wee et al. (2019) introduced a leave-one-region-out method to determine the most discriminative brain regions through a trial-and-error approach that assessed accuracy changes upon variable removal. Additionally, Fan et al. (2022) visualized connectograms that illustrated connectivity variations between diagnosis groups, while pooling scores were utilized as indicators of node importance in various studies. The BrainGNN model by Li X. et al. (2021) incorporated ROI-aware graph convolutional layers, enhancing the identification of significant brain regions through modified pooling techniques. Similarly, Cui et al. (2021) presented the BrainNNExplainer, which utilized shared masks to highlight critical connections in disease-specific brain networks, while Lee et al. (2021) combined GNNs with reinforcement learning to identify individually significant nodes. Finally, Gu et al. (2021) employed a GCN approach to evaluate node elimination impacts on experimental performance, facilitating the identification of node importance.

## 5 Limitations and potential breakthroughs

The literature reviewed in this survey demonstrates that GNN-based approaches are increasingly utilized for the diagnosis and early prediction of AD. Despite their promise, several technical challenges remain. These include limited sample size, graph construction methods, data scarcity, multimodal data integration, and generalization across domains. This section examines these challenges in detail and explores potential strategies to overcome them.

**Sample size:** Deep learning and GNN methods are typically data hungry-requiring large datasets-for effective model training. However, obtaining sufficient neuroimaging data poses significant challenges due to the resource-intensive nature of medical data collection, which often results in smaller datasets compared to fields like natural language processing or computer vision. This limitation has been a significant obstacle to the application of GNN methods in neuroimaging analysis (Xing et al., 2019; Wee et al., 2019). Traditional data augmentation techniques are commonly used to address this issue by increasing the size of training datasets (Shorten and Khoshgoftaar, 2019; Hao et al., 2024). However, these methods alone are often insufficient to mitigate overfitting in GNN models (Li X. et al., 2021). A promising alternative is transfer learning, which involves fine-tuning well-trained models from larger related datasets on smaller, disease-specific datasets. Integrating data augmentation with self-supervised learning presents another potential solution (Peng et al., 2022; Tang et al., 2022; Huang and Chung, 2020; Liu J. et al., 2020). Self-supervised learning can leverage the intrinsic structure of the data to improve model accuracy. For example, GNN models can be pre-trained using self-supervised loss functions and then fine-tuned for specific tasks, enhancing their performance on limited.

**Graph construction:** GNN methods leverage graph structures to learn feature representations from training data and make predictions for AD diagnosis. The choice of graph construction and representation methods, such as node definitions (e.g., ROIs, subjects, etc.), and edge construction (e.g., correlation, k-NN similarity, threshold, atlas/parcellation, etc.), is critical, as it directly impacts feature extraction and has a significant impact on GNN performance. Even minor modifications to parcellation or edge criteria can have a significant impact on results, causing reduced stability across study cohorts (Parisot et al., 2017, 2018; Ktena et al., 2018; Wee et al., 2019). Predefined graph methods, based on prior knowledge, have been widely employed; however, their effectiveness varies across datasets. This variability can compromise classification performance and introduce biases from irrelevant variables, such as sex, reducing diagnostic accuracy. Adaptive graph representation methods and multi-relation/hypergraph methods present a promising alternative by dynamically optimizing graph structures during training (Cui et al., 2023; Liu et al., 2023c; Kim, 2023; Huang and Chung, 2020). This approach aligns better with dataset-specific characteristics, reduces reliance on extensive hyperparameter tuning, and enhances overall performance.

**Data scarcity:** Missing data is a pervasive challenge in multimodal neuroimaging research. Subjects may lack certain modalities during data acquisition due to dropouts, or the low quality of specific modalities may necessitate their exclusion, leading to incomplete datasets (Pan et al., 2019). Conventional approaches often remove subjects without complete modality data, significantly reducing the training sample size and affecting diagnostic performance. Various data-imputation techniques exist; however, many focus on imputing hand-crafted feature values defined by domain experts to represent neuroimages. These features, however, often lack the discriminatory power required for accurate diagnosis and prognosis of AD. Recent studies (Pan et al., 2019, 2018) have explored approximating missing neuroimages, such as PET, using images from other modalities, like MRI. However, the interplay between imaging and non-imaging data has yet to be thoroughly examined. Future research could focus on developing sophisticated deep learning architectures capable of leveraging correlations across diverse data modalities, thereby enhancing the imputation of missing data and improving diagnostic performance.

**Multimodal data integration:** The advancement of neuroimaging technology allows for simultaneous analyses, yielding diverse disease-related features from various modalities. This multimodal approach offers a holistic view of brain morphology, structure, and function, thereby enhancing our understanding of individual conditions. While GNNs are inherently well-suited for handling multimodal data, their integration still poses several challenges. Data preprocessing and harmonization are critical, as varying scales and noise levels in imaging and genomic data can complicate integration. Additionally, the computational demands for processing extensive multimodal datasets may necessitate specialized hardware and software solutions (Xing et al., 2019; Zhou et al., 2022a; Choi et al., 2022). Current multimodal data fusion approaches fall into three categories. Data-level (early) fusion combines raw data from different modalities, while decision-level (late) fusion aggregates predictions from modality-specific classifiers. Intermediate fusion, an emerging strategy, employs advanced deep learning and GNN architectures to merge learned representations from multiple modalities at various abstraction levels (Hao et al., 2020). However, determining the optimal stage for integration within GNN architectures remains an open research question. A promising avenue is the use of MultiGraph approaches, which can effectively filter redundant information and synergize data across modalities. Moreover, different neuroimaging modalities capture information at distinct spatial and temporal scales. For example, fMRI provides second-scale temporal resolution, whereas structural MRI offers minute-scale spatial insights. Diagnosing AD requires a holistic understanding that encompasses both the spatial representation of affected brain regions and the temporal dynamics of disease progression (Zhang et al., 2018; Young et al., 2024). Although recent studies have investigated the spatial and temporal dimensions of AD pathology, they often focus exclusively on one aspect (Wang et al., 2019a,b). Future research should prioritize developing GNN frameworks capable of simultaneously integrating spatial and temporal data, paving the way for more robust and automated AD diagnosis.

**Generalization across domains:** Domain generalization presents a critical challenge in employing multimodal, multi-site data within GNN methods for AD diagnosis. When switching from single-site ADNI subsets to multi-site or external cohorts can significantly impact domain generalization, resulting drop in model performance due to domain shift (Wee et al., 2019). The recent interest in using multi-site data for AD diagnosis stems from the benefits of incorporating a large number of subjects from diverse imaging sites to study pathological changes in AD (Song et al., 2022). However, data collected across different sites often exhibit distribution bias, leading to inter-site heterogeneity arising from variations in acquisition protocols, scanning parameters, and subject demographics. Most existing methods assume that multi-site data come from the same distribution, which poses challenges for the generalization of GNN models. Consequently, building accurate and robust learning models that can handle heterogeneous multi-site data remains a significant challenge. To tackle inter-site heterogeneity, a promising research direction could involve utilizing adaptive learning and transferable features across multiple sites. Exploring domain generalization and domain adaptation as transfer learning strategies may help optimize GNN models for this purpose (Kumar et al., 2022; Wee et al., 2019; Li L. et al., 2022). For instance, harmonization and domain adaptation techniques can enable the training of GNN models using cross-site and cross-disease datasets.

**Explainability and interpretability:** Clinical adoption requires interpretability and explainability of the model outcomes. Despite improvements in interpretability, many GNN pipelines remain challenging and less interpretable and transparent than simpler models. In recent years, several studies (Kim et al., 2021; Zhou et al., 2022a; Tekkesinoglu and Pudas, 2024) incorporated GNN methods with post-hoc eXplainable Artificial Intelligence (XAI) modules (i.e., GNNExplainer, GRAD-CAM, attention maps, etc.), emphasizing the significance of linking model evidence to known neuroanatomy (such as salient ROIs) and to identify node/edge attribution in addition to the model performance.

## 6 Conclusion

This review provides a systematic and comprehensive analysis of the current state of research on the application of GNNs in neuroimaging for AD diagnosis and staging. We began by outlining the foundational principles of graphs and GNNs, including key components such as graph construction, convolution, pooling, and prediction. Subsequently, we evaluated diverse GNN applications across various data modalities, sample sizes, and diagnostic accuracy, emphasizing that multimodal GNN-based approaches have consistently demonstrated state-of-the-art performance in AD diagnosis. Key challenges were identified, including optimizing graph representations, addressing small sample size limitations, improving domain generalization, and enhancing multimodal data integration. To overcome these obstacles, we proposed several promising research directions, such as adaptive graph methods, transfer learning, advanced data fusion techniques, and frameworks that incorporate spatial and temporal data dimensions. The increasing understanding of AD pathophysiology, coupled with rapid advancements in GNN methodologies and the availability of extensive open-source datasets, provides a robust foundation for future exploration. Our findings underscore the significant potential of GNN-based models in improving the prediction, early diagnosis, and monitoring of AD progression. This review offers valuable insights to guide the integration of GNN methodologies with multimodal neuroimaging, ultimately aiming to refine diagnostic tools and enhance clinical decision-making.
