# Supplementary material for: Graph neural networks in Alzheimer's disease diagnosis: a review of unimodal and multimodal advances
Source: Front Neurosci. 2025 Sep 26;19:1623141. doi: 10.3389/fnins.2025.1623141 (PMC12511118; doi:10.3389/fnins.2025.1623141)
Supplement: Supplementary file 1 [file Data_Sheet_1.pdf]

Supplementary File

Graph Neural Networks in Alzheimer's Disease Diagnosis: A Review of Unimodal and Multimodal Advances

Shahzad Ali, Michele Piana, Matteo Pardini, and Sara Garbarino

1 SUPPLEMENTARY DATA

Table S1. GitHub Repositories for Open-Source Code in Reviewed Studies

Reference	GitHub Repository
Parisot et al. (2018)	https://github.com/parisots/population-gcn
Ktena et al. (2018)	https://github.com/sk1712/gcn_metric_learning
Jiang et al. (2020)	https://github.com/haojiang1/hi-GCN
Song et al. (2021)	https://github.com/SJTUBME-QianLab/AutoMetricGNN
Kim et al. (2021)	https://github.com/JaesikKim/temporal-GNN
Yao et al. (2021)	https://github.com/Brain03Yao/MMTGCN
Wen et al. (2022)	https://github.com/GuangqiWen/MVS-GCN
Song et al. (2022)	https://github.com/Xuegang-S
Zheng et al. (2022)	https://github.com/SsGood/MMGL
Li et al. (2022a)	https://github.com/llt1836/TE-HI-GCN
McCombe et al. (2022)	https://github.com/mac-n/Clustering-GNN
Qu et al. (2023)	https://github.com/Zongshuaiqu/UNB-GCN
Hao et al. (2024)	https://github.com/bob-lee-student/WHGCN
Tekkesinoglu and Pudas (2024)	https://github.com/suletekkesinoglu/GCN_XAI_ADNI

Note: Only studies with publicly available code on GitHub are listed (accessed on 18 August 2025).

Table S2: Distribution of Studies and Performance Metrics by GNN Architecture and Task.

GNN Architecture	NC vs. AD	NC vs. MCI	MCI vs. AD	References
**a) Performance of GNN Architectures in Unimodal Studies**				
Spectral-ChebNet	89.17±2.44$_{[85.80-91.51]}$	71.12±14.63$_{[51.80-93.00]}$	80.50±1.30$_{[79.20-81.80]}$	Wee et al. (2019); Ktena et al. (2018); Kumar et al. (2022); Zuo and Kamata (2023); Guo et al. (2019)
Spectral-GCN	86.58±6.87$_{[71.30-96.18]}$	76.68±5.54$_{[73.37-88.90]}$	75.77±12.69$_{[53.50-94.70]}$	Kim et al. (2021); Zhu et al. (2021); Peng et al. (2022); Zhang et al. (2023a); Aafiya and Jeyachidra (2024); Liu et al. (2024); Hao et al. (2024); Liu et al. (2020b); Gu et al. (2021); Lee et al. (2021); Tang et al. (2022); Mei et al. (2022); Wen et al. (2022); Qin et al. (2022); Liu et al. (2023b,c); Song et al. (2019); Klepl et al. (2022)
Spectral-Other	86.20±0.00$_{[86.20-86.20]}$	75.27±10.23$_{[63.90-93.50]}$	—	Fan et al. (2023); Zhao et al. (2019); Li et al. (2023c); Cai et al. (2023)
Spatial-GIN	90.44±0.00$_{[90.44-90.44]}$	—	—	Wang et al. (2023b)
Spatial-Other	79.83±9.22$_{[67.22-89.00]}$	—	—	Fan et al. (2022)
ST-RNN	80.23±6.66$_{[72.70-88.90]}$	78.60±0.00$_{[78.60-78.60]}$	—	Liu et al. (2023a)
ST-CNN	91.10±0.00$_{[91.10-91.10]}$	—	—	Shan et al. (2022)
ST-Other	99.16±0.00$_{[99.16-99.16]}$	—	—	Wang et al. (2023a)

Continued on next page

Table S2 (continued)

GNN Architecture	NC vs. AD	NC vs. MCI	MCI vs. AD	References
MultiGraph-GCN	—	$86.75 \pm 3.35_{[83.40\text{-}90.10]}$	—	Yao et al. (2021); Cui et al. (2023)

b) Performance of GNN Architectures in Multimodal Studies

GNN Architecture	NC vs. AD	NC vs. MCI	MCI vs. AD	References
Spectral-ChebNet	$95.44 \pm 1.18_{[93.88\text{-}96.94]}$	$83.89 \pm 3.12_{[80.00\text{-}88.18]}$	$83.33 \pm 0.00_{[83.33\text{-}83.33]}$	Parisot et al. (2018); Zhu et al. (2021); Kazi et al. (2019b,a); Huang and Chung (2020); Zhang et al. (2023c,b)
Spectral-GCN	$89.49 \pm 7.58_{[77.20\text{-}99.30]}$	$91.29 \pm 3.86_{[84.10\text{-}98.00]}$	$86.41 \pm 5.81_{[78.50\text{-}94.60]}$	Kim (2023); Yu et al. (2019); Jiang et al. (2020); Subaramya et al. (2022); Lin et al. (2023); Li et al. (2023a); McCombe et al. (2022); Song et al. (2022); Liu et al. (2020a); Qu et al. (2023); Tekkesinoglu and Pudas (2024); Zhou et al. (2022a,b); Zhang et al. (2023d)
Spectral-Other	$94.84 \pm 1.23_{[93.61\text{-}96.06]}$	$89.62 \pm 7.91_{[76.78\text{-}98.25]}$	$89.73 \pm 5.56_{[82.09\text{-}95.15]}$	Bi et al. (2023); Salim and Hamza (2024); Meng and Zhang (2023); Li et al. (2022b); Zhang et al. (2022)
Spatial-GraphSAGE	$93.72 \pm 5.01_{[88.71\text{-}98.72]}$	$88.50 \pm 6.13_{[79.68\text{-}95.83]}$	$86.33 \pm 3.63_{[82.70\text{-}89.96]}$	Song et al. (2021); Zheng et al. (2022); Tian et al. (2023); Chen et al. (2024)
Spatial-GAT	$96.00 \pm 0.00_{[96.00\text{-}96.00]}$	—	—	Choi et al. (2022)
Spatial-Other	$92.55 \pm 4.55_{[88.00\text{-}97.10]}$	$92.20 \pm 5.00_{[87.21\text{-}97.20]}$	$92.40 \pm 0.00_{[92.40\text{-}92.40]}$	Yang et al. (2023); Li et al. (2023b)
ST-RNN	—	$85.06 \pm 5.34_{[79.73\text{-}90.40]}$	$86.70 \pm 0.00_{[86.70\text{-}86.70]}$	Li et al. (2022a); Yang et al. (2022); Xing et al. (2019)
ST-Other	$93.50 \pm 0.00_{[93.50\text{-}93.50]}$	—	—	Chhabra et al. (2023)
MultiGraph-GCN	—	$87.89 \pm 3.94_{[84.80\text{-}93.46]}$	—	Lei et al. (2023); Guo et al. (2023)

Note. Values are reported as mean±SD$_{[range]}$

NC vs. MCI accuracies were aggregated from tasks such as NC/MCI, NC/EMCI, and NC/LMCI. Similarly, MCI vs. AD accuracies were summarized from tasks including EMCI/AD, MCI/AD, and LMCI/AD.

REFERENCES

Aafiya and Jeyachidra (2024). Advancing alzheimer's disease detection harnessing graph convolutional networks for enhanced early identification. *International Research Journal on Advanced Engineering Hub (IRJAEH)* 2, 1019–1027. doi:10.47392/IRJAEH.2024.0142

Bi, X.-A., Chen, K., Jiang, S., Luo, S., Zhou, W., Xing, Z., et al. (2023). Community graph convolution neural network for alzheimer's disease classification and pathogenetic factors identification. *IEEE Transactions on Neural Networks and Learning Systems*

Cai, H., Zhou, Z., Yang, D., Wu, G., and Chen, J. (2023). Discovering brain network dysfunction in alzheimer's disease using brain hypergraph neural network. In *International Conference on Medical Image Computing and Computer-Assisted Intervention* (Springer), 230–240

Chen, K., Weng, Y., Hosseini, A. A., Dening, T., Zuo, G., and Zhang, Y. (2024). A comparative study of gnn and mlp based machine learning for the diagnosis of alzheimer's disease involving data synthesis. *Neural Networks* 169, 442–452

Chhabra, G. S., Guru, A., Rajput, B. J., Dewangan, L., and Swarnkar, S. K. (2023). Multimodal neuroimaging for early alzheimer's detection: A deep learning approach. In *2023 14th International Conference on Computing Communication and Networking Technologies (ICCCNT)* (IEEE), 1–5

Choi, I., Wu, G., and Kim, W. H. (2022). How much to aggregate: Learning adaptive node-wise scales on graphs for brain networks. In *Medical Image Computing and Computer Assisted Intervention – MICCAI 2022*, eds. L. Wang, Q. Dou, P. T. Fletcher, S. Speidel, and S. Li (Cham: Springer Nature Switzerland), 376–385

Cui, W., Ma, Y., Ren, J., Liu, J., Ma, G., Liu, H., et al. (2023). Personalized functional connectivity based spatio-temporal aggregated attention network for mci identification. *IEEE Transactions on Neural Systems and Rehabilitation Engineering* 31, 2257–2267

Fan, C.-C., Yang, H., Peng, L., Zhou, X.-H., Ni, Z.-L., Zhou, Y.-J., et al. (2022). Bgl-net: A brain-inspired global-local information fusion network for alzheimer's disease based on smri. *IEEE Transactions on Cognitive and Developmental Systems* 15, 1161–1169

Fan, C.-C., Yang, H., Zhang, C., Peng, L., Zhou, X., Liu, S., et al. (2023). Graph reasoning module for alzheimer's disease diagnosis: A plug-and-play method. *IEEE Transactions on Neural Systems and Rehabilitation Engineering* 31, 4773–4780

Gu, P., Xu, X., Luo, Y., Wang, P., and Lu, J. (2021). Bcn-gcn: A novel brain connectivity network classification method via graph convolution neural network for alzheimer's disease. In *Neural Information Processing: 28th International Conference, ICONIP 2021, Sanur, Bali, Indonesia, December 8–12, 2021, Proceedings, Part I 28* (Springer), 657–668

Guo, J., Qiu, W., Li, X., Zhao, X., Guo, N., and Li, Q. (2019). Predicting alzheimer's disease by hierarchical graph convolution from positron emission tomography imaging. In *2019 IEEE international conference on big data (big data)* (IEEE), 5359–5363

Guo, R., Tian, X., Lin, H., McKenna, S., Li, H.-D., Guo, F., et al. (2023). Graph-based fusion of imaging, genetic and clinical data for degenerative disease diagnosis. *IEEE/ACM Transactions on Computational Biology and Bioinformatics*

Hao, X., Li, J., Ma, M., Qin, J., Zhang, D., Liu, F., et al. (2024). Hypergraph convolutional network for longitudinal data analysis in alzheimer's disease. *Computers in Biology and Medicine* 168, 107765

Huang, Y. and Chung, A. C. (2020). Edge-variational graph convolutional networks for uncertainty-aware disease prediction. In *Medical Image Computing and Computer Assisted Intervention–MICCAI 2020: 23rd International Conference, Lima, Peru, October 4–8, 2020, Proceedings, Part VII 23* (Springer), 562–572

Jiang, H., Cao, P., Xu, M., Yang, J., and Zaiane, O. (2020). Hi-gcn: A hierarchical graph convolution network for graph embedding learning of brain network and brain disorders prediction. *Computers in Biology and Medicine* 127, 104096

Kazi, A., Shekarforoush, S., Arvind Krishna, S., Burwinkel, H., Vivar, G., Kortüm, K., et al. (2019a). Inceptiongcn: receptive field aware graph convolutional network for disease prediction. In *Information Processing in Medical Imaging: 26th International Conference, IPMI 2019, Hong Kong, China, June 2–7, 2019, Proceedings 26* (Springer), 73–85

Kazi, A., Shekarforoush, S., Arvind Krishna, S., Burwinkel, H., Vivar, G., Wiestler, B., et al. (2019b). Graph convolution based attention model for personalized disease prediction. In *Medical Image Computing and Computer Assisted Intervention–MICCAI 2019: 22nd International Conference, Shenzhen, China, October 13–17, 2019, Proceedings, Part IV 22* (Springer), 122–130

Kim, M., Kim, J., Qu, J., Huang, H., Long, Q., Sohn, K.-A., et al. (2021). Interpretable temporal graph neural network for prognostic prediction of alzheimer's disease using longitudinal neuroimaging data. In *2021 IEEE International Conference on Bioinformatics and Biomedicine (BIBM)* (IEEE), 1381–1384

Kim, S. Y. (2023). Personalized explanations for early diagnosis of alzheimer's disease using explainable graph neural networks with population graphs. *Bioengineering* 10, 701

Klepl, D., He, F., Wu, M., Blackburn, D. J., and Sarrigiannis, P. (2022). Eeg-based graph neural network classification of alzheimer's disease: An empirical evaluation of functional connectivity methods. *IEEE Transactions on Neural Systems and Rehabilitation Engineering* 30, 2651–2660

Ktena, S. I., Parisot, S., Ferrante, E., Rajchl, M., Lee, M., Glocker, B., et al. (2018). Metric learning with spectral graph convolutions on brain connectivity networks. *NeuroImage* 169, 431–442

Kumar, A., Balaji, V., Chandrashekar, M., Dukkipati, A., and Vadhiyar, S. (2022). Graph convolutional neural networks for alzheimer's classification with transfer learning and hpc methods. In *2022 IEEE International Parallel and Distributed Processing Symposium Workshops (IPDPSW)* (IEEE), 186–195

Lee, J., Ko, W., Kang, E., Suk, H.-I., Initiative, A. D. N., et al. (2021). A unified framework for personalized regions selection and functional relation modeling for early mci identification. *NeuroImage* 236, 118048

Lei, B., Zhu, Y., Yu, S., Hu, H., Xu, Y., Yue, G., et al. (2023). Multi-scale enhanced graph convolutional network for mild cognitive impairment detection. *Pattern Recognition* 134, 109106. doi:https://doi.org/10.1016/j.patcog.2022.109106

Li, F., Wang, Z., Guo, Y., Liu, C., Zhu, Y., Zhou, Y., et al. (2023a). Dynamic dual-graph fusion convolutional network for alzheimer's disease diagnosis. In *2023 IEEE International Conference on Image Processing (ICIP)* (IEEE), 675–679

Li, L., Jiang, H., Wen, G., Cao, P., Xu, M., Liu, X., et al. (2022a). Te-hi-gcn: An ensemble of transfer hierarchical graph convolutional networks for disorder diagnosis. *Neuroinformatics* , 1–23

Li, W., Zhao, J., Shen, C., Zhang, J., Hu, J., Xiao, M., et al. (2022b). Regional brain fusion: Graph convolutional network for alzheimer's disease prediction and analysis. *Frontiers in Neuroinformatics* 16. doi:10.3389/fninf.2022.886365

Li, Y., Yang, B., Pan, D., Zeng, A., Wu, L., and Yang, Y. (2023b). Early diagnosis of alzheimer's disease based on multimodal hypergraph attention network. In *2023 IEEE International Conference on Multimedia and Expo (ICME)* (IEEE), 192–197

Li, Y., Zou, Y., Guo, H., Yang, Y., Li, N., Li, L., et al. (2023c). Identification of mild cognitive impairment based on quadruple gcn model constructed with multiple features from higher-order brain connectivity. *Expert Systems with Applications* 230, 120575

Lin, X., Geng, Y., Zhao, J., Cheng, D., Zhang, X., and Liang, H. (2023). Multi-modal medical image classification method combining graph convolution neural networks. In *2023 26th International Conference on Computer Supported Cooperative Work in Design (CSCWD)* (IEEE), 199–206

Liu, J., Tan, G., Lan, W., and Wang, J. (2020a). Identification of early mild cognitive impairment using multi-modal data and graph convolutional networks. *BMC bioinformatics* 21, 1–12

Liu, M., Zhang, H., Shi, F., and Shen, D. (2023a). Hierarchical graph convolutional network built by multiscale atlases for brain disorder diagnosis using functional connectivity. *IEEE Transactions on Neural Networks and Learning Systems*

Liu, T., Liu, F., Wan, Y., Hu, R., Zhu, Y., and Li, L. (2024). Hierarchical graph learning with convolutional network for brain disease prediction. *Multimedia Tools and Applications* 83, 46161–46179

Liu, X., Li, J., and Cao, P. (2020b). S-gcn: A siamese spectral graph convolutions on brain connectivity networks. In *The Fourth International Symposium on Image Computing and Digital Medicine*. 46–48

Liu, Y., Liu, M., Zhang, Y., and Shen, D. (2023b). Development and fast transferring of general connectivity-based diagnosis model to new brain disorders with adaptive graph meta-learner. In *International Conference on Medical Image Computing and Computer-Assisted Intervention* (Springer), 99–108

Liu, Y., Liu, M., Zhang, Y., and Shen, D. (2023c). Learning hierarchical-order functional connectivity networks for mild cognitive impairment diagnosis. In *2023 IEEE 20th International Symposium on Biomedical Imaging (ISBI)* (IEEE), 1–5

McCombe, N., Bamrah, J., Sanchez-Bornot, J. M., Finn, D. P., McClean, P. L., Wong-Lin, K., et al. (2022). Alzheimer's disease classification using cluster-based labelling for graph neural network on heterogeneous data. *Healthcare Technology Letters* 9, 102–109

Mei, L., Liu, M., Bian, L., Zhang, Y., Shi, F., Zhang, H., et al. (2022). Modular graph encoding and hierarchical readout for functional brain network based emci diagnosis. In *MICCAI Workshop on Imaging Systems for GI Endoscopy* (Springer), 69–78

Meng, L. and Zhang, Q. (2023). Research on early diagnosis of alzheimer's disease based on dual fusion cluster graph convolutional network. *Biomedical Signal Processing and Control* 86, 105212

Parisot, S., Ktena, S. I., Ferrante, E., Lee, M., Guerrero, R., Glocker, B., et al. (2018). Disease prediction using graph convolutional networks: application to autism spectrum disorder and alzheimer's disease. *Medical image analysis* 48, 117–130

Peng, L., Wang, N., Dvornek, N., Zhu, X., and Li, X. (2022). Fedni: Federated graph learning with network inpainting for population-based disease prediction. *IEEE Transactions on Medical Imaging* 42, 2032–2043

Qin, Z., Liu, Z., and Zhu, P. (2022). Aiding alzheimer's disease diagnosis using graph convolutional networks based on rs-fmri data. In *2022 15th International Congress on Image and Signal Processing, BioMedical Engineering and Informatics (CISP-BMEI)* (IEEE), 1–7

Qu, Z., Yao, T., Liu, X., and Wang, G. (2023). A graph convolutional network based on univariate neurodegeneration biomarker for alzheimer's disease diagnosis. *IEEE Journal of Translational Engineering in Health and Medicine* 11, 405–416

Salim, I. and Hamza, A. B. (2024). Classification of developmental and brain disorders via graph convolutional aggregation. *Cognitive Computation* 16, 701–716

Shan, X., Cao, J., Huo, S., Chen, L., Sarrigiannis, P. G., and Zhao, Y. (2022). Spatial–temporal graph convolutional network for alzheimer classification based on brain functional connectivity imaging of electroencephalogram. *Human Brain Mapping* 43, 5194–5209

Song, T.-A., Chowdhury, S. R., Yang, F., Jacobs, H., El Fakhri, G., Li, Q., et al. (2019). Graph convolutional neural networks for alzheimer's disease classification. In *2019 IEEE 16th international symposium on biomedical imaging (ISBI 2019)* (IEEE), 414–417

Song, X., Mao, M., and Qian, X. (2021). Auto-metric graph neural network based on a meta-learning strategy for the diagnosis of alzheimer's disease. *IEEE Journal of Biomedical and Health Informatics* 25, 3141–3152

Song, X., Zhou, F., Frangi, A. F., Cao, J., Xiao, X., Lei, Y., et al. (2022). Multicenter and multichannel pooling gcn for early ad diagnosis based on dual-modality fused brain network. *IEEE Transactions on Medical Imaging* 42, 354–367

Subaramya, S., Kokul, T., Nagulan, R., and Pinidiyaarachchi, U. (2022). Graph neural network based alzheimer's disease classification using structural brain network. In *2022 22nd International Conference on Advances in ICT for Emerging Regions (ICTer)* (IEEE), 1–6

Tang, H., Ma, G., Guo, L., Fu, X., Huang, H., and Zhan, L. (2022). Contrastive brain network learning via hierarchical signed graph pooling model. *IEEE transactions on neural networks and learning systems*

Tekkesinoglu, S. and Pudas, S. (2024). Explaining graph convolutional network predictions for clinicians—an explainable ai approach to alzheimer's disease classification. *Frontiers in Artificial Intelligence* 6, 1334613

Tian, X., Liu, Y., Wang, L., Zeng, X., Huang, Y., and Wang, Z. (2023). An extensible hierarchical graph convolutional network for early alzheimer's disease identification. *Computer Methods and Programs in Biomedicine* 238, 107597

Wang, X., Xin, J., Wang, Z., Chen, Q., and Wang, Z. (2023a). An evolving graph convolutional network for dynamic functional brain network. *Applied Intelligence* 53, 13261–13274

Wang, Z., Lin, Z., Li, S., Wang, Y., Zhong, W., Wang, X., et al. (2023b). Dynamic multi-task graph isomorphism network for classification of alzheimer's disease. *Applied Sciences* 13, 8433

Wee, C.-Y., Liu, C., Lee, A., Poh, J. S., Ji, H., Qiu, A., et al. (2019). Cortical graph neural network for ad and mci diagnosis and transfer learning across populations. *NeuroImage: Clinical* 23, 101929

Wen, G., Cao, P., Bao, H., Yang, W., Zheng, T., and Zaiane, O. (2022). Mvs-gcn: A prior brain structure learning-guided multi-view graph convolution network for autism spectrum disorder diagnosis. *Computers in biology and medicine* 142, 105239

Xing, X., Li, Q., Wei, H., Zhang, M., Zhan, Y., Zhou, X. S., et al. (2019). Dynamic spectral graph convolution networks with assistant task training for early mci diagnosis. In *International Conference on Medical Image Computing and Computer-Assisted Intervention* (Springer), 639–646

Yang, F., Wang, H., Wei, S., Sun, G., Chen, Y., and Tao, L. (2023). Multi-model adaptive fusion-based graph network for alzheimer's disease prediction. *Computers in Biology and Medicine* 153, 106518

Yang, Y., Guo, X., Chang, Z., Ye, C., Xiang, Y., and Ma, T. (2022). Multi-modal dynamic graph network: Coupling structural and functional connectome for disease diagnosis and classification. In *2022 IEEE International Conference on Bioinformatics and Biomedicine (BIBM)*. 1343–1349. doi:10.1109/BIBM55620.2022.9995642

Yao, D., Sui, J., Wang, M., Yang, E., Jiaerken, Y., Luo, N., et al. (2021). A mutual multi-scale triplet graph convolutional network for classification of brain disorders using functional or structural connectivity. *IEEE transactions on medical imaging* 40, 1279–1289

Yu, S., Yue, G., Elazab, A., Song, X., Wang, T., and Lei, B. (2019). Multi-scale graph convolutional network for mild cognitive impairment detection. In *Graph Learning in Medical Imaging: First International Workshop, GLMI 2019, Held in Conjunction with MICCAI 2019, Shenzhen, China, October 17, 2019, Proceedings 1* (Springer), 79–87

Zhang, H., Song, R., Wang, L., Zhang, L., Wang, D., Wang, C., et al. (2022). Classification of brain disorders in rs-fmri via local-to-global graph neural networks. *IEEE transactions on medical imaging* 42, 444–455

Zhang, J., He, X., Qing, L., Chen, X., Liu, Y., and Chen, H. (2023a). Multi-relation graph convolutional network for alzheimer's disease diagnosis using structural mri. *Knowledge-Based Systems* 270, 110546

Zhang, Y., Cai, Q., He, X., Ren, X., Zhang, L., and Liu, Y. (2023b). A joint cnn-gnn framework for early diagnosis of ad using multi-source multi-modal data. In *2023 IEEE International Conference on Bioinformatics and Biomedicine (BIBM)* (IEEE), 2407–2411

Zhang, Y., He, X., Chan, Y. H., Teng, Q., and Rajapakse, J. C. (2023c). Multi-modal graph neural network for early diagnosis of alzheimer's disease from smri and pet scans. *Computers in Biology and Medicine* 164, 107328

Zhang, Y., Qing, L., He, X., Zhang, L., Liu, Y., and Teng, Q. (2023d). Population-based gcn method for diagnosis of alzheimer's disease using brain metabolic or volumetric features. *Biomedical Signal Processing and Control* 86, 105162

Zhao, X., Zhou, F., Ou-Yang, L., Wang, T., and Lei, B. (2019). Graph convolutional network analysis for mild cognitive impairment prediction. In *2019 IEEE 16th International Symposium on Biomedical Imaging (ISBI 2019)* (IEEE), 1598–1601

Zheng, S., Zhu, Z., Liu, Z., Guo, Z., Liu, Y., Yang, Y., et al. (2022). Multi-modal graph learning for disease prediction. *IEEE Transactions on Medical Imaging* 41, 2207–2216

Zhou, H., He, L., Zhang, Y., Shen, L., and Chen, B. (2022a). Interpretable graph convolutional network of multi-modality brain imaging for alzheimer's disease diagnosis. In *2022 IEEE 19th International Symposium on Biomedical Imaging (ISBI)* (IEEE), 1–5

Zhou, H., Zhang, Y., Chen, B. Y., Shen, L., and He, L. (2022b). Sparse interpretation of graph convolutional networks for multi-modal diagnosis of alzheimer's disease. In *International Conference on Medical Image Computing and Computer-Assisted Intervention* (Springer), 469–478

Zhu, Y., Song, X., Qiu, Y., Zhao, C., and Lei, B. (2021). Structure and feature based graph u-net for early alzheimer's disease prediction. In *Multimodal Learning for Clinical Decision Support: 11th International Workshop, ML-CDS 2021, Held in Conjunction with MICCAI 2021, Strasbourg, France, October 1, 2021, Proceedings 11* (Springer), 93–104

Zuo, Z. and Kamata, S.-I. (2023). Ad/mci classification using dmn connectivity networks-based hgcn with attention. In *2023 IEEE 6th International Conference on Pattern Recognition and Artificial Intelligence (PRAI)* (IEEE), 1147–1154
